# NMR Based Real-Time Analysis of Exometabolites Decodes the Mechanism of Action of Antibacterial Molecules, Nanoparticles, and Materials

**DOI:** 10.1021/acs.analchem.5c06983

**Published:** 2026-02-23

**Authors:** Simona Tomaselli, Roberto K. Salinas, Michela Alfè, Valentina Gargiulo, Simona Losio, Laura Ragona

**Affiliations:** 1 366612 Istituto di Scienze e Tecnologie Chimiche (SCITEC), CNR, Via A. Corti 12, 20133 Milan, Italy; 2 Institute of Chemistry, 28133 University of São Paulo, Av. Prof. Lineu Prestes, 748, 05508-000 São Paulo, Brazil; 3 Istituto di scienze e tecnologie per l’energia e la mobilità sostenibili (STEMS), CNR,Via G. Marconi 4, 80125 Naples, Italy

## Abstract

Understanding the mechanism of action of antimicrobial agents is critical for guiding the development of new drugs to overcome antimicrobial resistance. We present a label-free NMR-based approach to characterize the mechanism of action of antibacterial compounds and materials by the analysis of metabolite secretion kinetics. The method (KINEXO, KINetics of EXOmetabolites) is set up using *Escherichia coli* and *Staphylococcus aureus* as representative Gram-negative and Gram-positive model organisms. By monitoring the real-time production of key secreted metabolites (acetate, formate, lactate, ethanol, pyruvate, succinate) in response to antimicrobial treatment and analyzing the secretion kinetics, we can classify the agents’ mechanisms of action. We validate KINEXO using agents with well-characterized mechanism of action (kanamycin, ampicillin, irgasan, caprylic acid, graphene-like nanoparticles, and a functionalized silicon material), and we further apply it to silver nanoparticles, whose mechanism of action remains under debate. Agents that perturb the cell envelope reduce secretion rates while maintaining end-point metabolite concentrations with only moderate lag phase extension. In contrast, agents that act on intracellular pathways drastically prolong lag phases and reduce both secretion rates and end-point concentrations. When plotted in 3D parameter space (exometabolite secretion lag time, secretion rate, end-point concentration), antibacterial agents cluster according to their mode of action, offering a mechanistically informative phenotypic readout. This platform provides a generalizable and robust analytical framework for rapid antimicrobial profiling and mechanism-based screening of novel bioactive agents.

## Graphical abstract

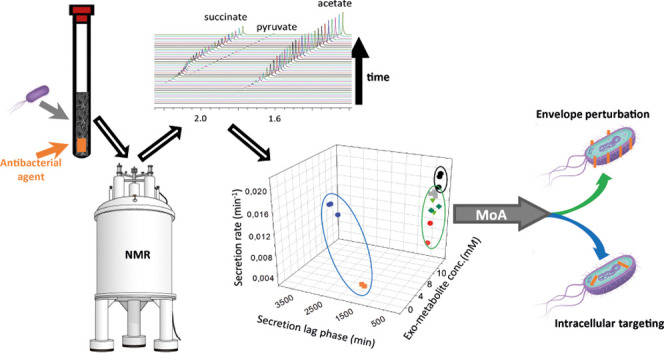

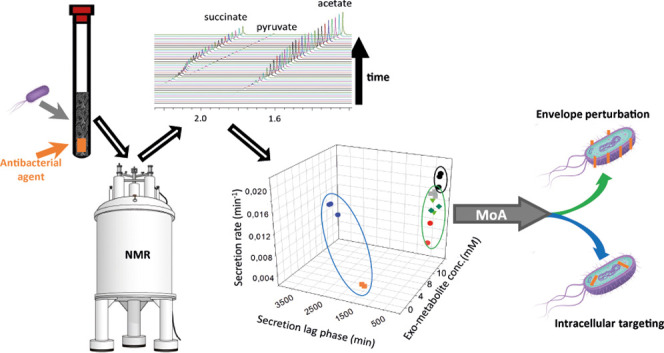

## Introduction

To design new materials, nanoparticles, and drugs with antibacterial activity, it is essential to thoroughly understand their mechanism of action (MoA) against the target bacteria. Understanding MoA is crucial not only for guiding the design of these agents but also to assess the risks associated with their application and to develop new formulations that combine effective antibacterial activity with a few secondary adverse effects, such as antimicrobial resistance. Characterization of antibacterial MoAs of a material, nanoparticle, or molecule requires a combined experimental and theoretical approach, which may include various chemical, physical, and biological techniques. Generally, such a study starts with the definition of the antibacterial agent activity supported by viability tests (colony forming units (CFU) counts, minimum inhibitory concentration (MIC) and minimum bactericidal concentration determination (MBC), inhibition zones, etc.). The study of MoA is often carried out using a series of strategies, such as analysis of cellular morphology by electron microscopy (SEM/TEM) to detect physical damage to the bacterial membrane, or to study the aggregation of nanoparticles on the membrane surface.^1–3^ Atomic force microscopy (AFM) can be used to study the formation of biofilms on the surface of the material in contact with bacteria.^4,5^ Membrane integrity can be evaluated by measurements of permeability, the cytoplasmic h-galactosidase activity, or cytoplasmic membrane depolarization^6^ or by flow cytometry using SYBR Green and propidium iodide,^7^ to detect intracellular protein, DNA and ions leakage.^8^ Biochemical assays typically used to investigate MoA of antibacterial agents include (i) detection of reactive oxygen species using multiple techniques including colorimetric assays, immunoblotting, and immunofluorescence;^9^ (ii) detection of the metabolic state of living cells through the MTT (3-(4,5-dimethylthiazol-2-yl)-2,5-diphenyltetrazolium bromide) tetrazolium assay;^10^ (iii) enzymatic studies;^11–13^ (iv) omics analysis to understand changes in metabolic state and protein/RNA levels upon contact with the antibacterial agent/material.^14,15^ Most of the strategies mentioned above address the end points of metabolic processes altered by antibacterial treatment, whereas omics studies give a more specific picture of how an antibacterial agent affects specific metabolic pathways, altering cellular physiology. In particular, the assessment of metabolite levels over time (time-resolved metabolomics) is increasingly used for analytical characterization and monitoring the dynamics of metabolic pathways,^16,17^ revealing subtle changes induced by the presence of environmental stress factors such as antibacterial agents.^18–20^ The dynamics of intra- and extracellular metabolites is generally followed by analytical techniques such as time-resolved 1D and 2D NMR^16,21–23^ and mass spectrometry approaches.^24–26^

It has been proposed that metabolomics can be very useful for studying the modes of action of antibiotics.^27,28,11^ In particular, extracellular metabolites (exometabolites), the set of low molecular weight compounds secreted in the extracellular environment to allow cells to maintain their biochemical balance, are key factors in intercellular communication,^29^ and their altered profiles can easily reveal the cellular responses to the presence of environmental stresses,^30,31^ such as antibacterial agents. Nearly all microbial cells secrete metabolites, and we previously showed that NMR allows us to easily monitor changes in the concentrations of metabolites in real time by recording a time series of 1D ^1^H spectra on samples of bacterial cells growing in the NMR tube.^22^ The limited number of secreted molecules makes the assignment step straightforward and the changes occurring over time in the exometabolites chemical shift and signal intensity mirror their secretion kinetics. Exometabolite analysis eliminates the need for complex sample manipulation typically needed to derive intracellular metabolites profiles (quenching, extraction of metabolites from cells and processing), thus avoiding time-consuming steps and increasing data reproducibility.^29^

Here, we discuss the key factors to obtain highly reproducible secretion curves and demonstrate that quantitative analysis in terms of exometabolites’ secretion rates, lag phases, and end-point concentrations is a valuable tool for the classification of the MoA of small molecules and more generally of nanoparticles and materials. We propose an analytical protocol, KINEXO, guiding the discrimination between antibacterial agents entering the bacterial cells and targeting intracellular pathways from those perturbing the cell wall. KINEXO, built on the time-resolved quantitative assessment of exometabolome, enables scalable, label-free, robust analytical framework for rapid antimicrobial profiling of novel bioactive agents.

## Experimental Section

### Culture Medium for *E. coli* and *S. aureus*

Bacterial strains, *E. coli* (ATCC 11229) and *S. aureus* (ATCC 6538), were purchased from Biogenetics Diagnostics srl. Rich media (r.m.), peptone water/yeast agar, and BHI (brain heart infusion)/BHI agar, used for *E. coli* and *S. aureus*, respectively, were purchased from Merck. Minimal growth medium (m.m.) for *E. coli* is a modified MM9 adapted for experimental needs containing 0.02 M NaCl, 0.02 M NH_4_Cl, 0.022 M KH_2_PO_4_, 0.048 M Na_2_HPO_4_, 2 mM MgSO_4_, 0.1 mM ZnSO_4_, 0.2 mM FeCl_3_, and 0.8% glucose. *S. aureus* was initially grown in the minimal medium proposed by Machado and co-workers,^32^ containing only three amino acids (R, C, P) but using 100 mg of each amino acid instead of 200 mg as in the original method. However, even after 24 h at 37 °C, *S. aureus* cells had not grown enough to show any detectable changes in the secreted metabolite concentrations. For this reason, *S. aureus* cells were grown in a diluted rich medium: 30 mM potassium phosphate buffer (KPi) pH 7, enriched with 5% v/v BHI (see Supporting Information).

### Dilution Scheme

Bacteria were inoculated in rich medium, r.m., from a glycerol stock stored at −80 °C. This preinoculum was kept at 37 °C for 24 h (Scheme S1, step A). Minimal medium, m.m., was inoculated with this overnight (o.n.) culture in r.m. at a 1:10^4^ dilution, and cultured at 37 °C for 18 or 24 h (Scheme S1, step B). To select the best culture cultivation time in step B, the kinetic parameters of secreted metabolites were compared (see Supporting Information) and 18 h preculture time was selected. This intermediate step B in m.m. was necessary to allow bacteria to adapt to the m.m. and to reach the exponential growth phase in a reasonable time lapse with high reproducibility. A final 1:1000 dilution was performed to inoculate cell cultures in fresh media (Scheme S1, step C), which were then transferred to the NMR tube to record NMR spectra. The same dilution steps were performed for both the *E. coli* and *S. aureus* cell cultures. Following this scheme, the NMR sample contained an average of 10^5^ cells at the beginning of the NMR experiment, which increased up to roughly 10^10^ and 10^8^ cells after 18 h in the NMR tube for *E. coli* and *S. aureus*, respectively.

### NMR Experiments

NMR samples were prepared considering an 800 μL final volume containing 10% D_2_O and TSP 0.2 mM. The latter was used as an internal standard for metabolite quantification and as a chemical shift reference. Cell cultures were introduced in NMR tubes previously sterilized at 121 °C in an autoclave. 1D ^1^H NMR experiments were acquired at 37 °C 500 MHz (Bruker Avance III), equipped with a room temperature probe, and at 600 MHz (Bruker NEO), equipped with a PRODIGY cryoprobe. Water suppression was achieved by using gradient excitation sculpting. A series of NMR spectra, acquired employing a relaxation delay (*D*_1_) of 2.5 s for a total acquisition time of 3 min, were used to derive kinetic parameters. Secretion curves were obtained by integrating selected resonances, free from overlap, and plotting integrals as a function of time. Spectra with longer *D*_1_ of 25 s were recorded when the secretion curves reached a plateau to derive end-point metabolite concentrations. Spectra used to test integrity of the samples after 24 h in the NMR tube were collected with a *D*_1_ of 25s, 128 scans for a total acquisition time of 1 h.

Spectra were processed and automatically integrated with Topspin v 3.6 software. Protons selected as probes of metabolite concentration over time are highlighted in Figure S1. The progressive accumulation of acid metabolites lowered the pH from 7.0 to 4.5 and induced changes in the chemical shift during NMR data acquisition. Peaks influenced by pH, such as formate, acetate, and succinate, are well isolated and thus readily integrated by automated routines by setting the proper chemical shift range. For the integration of *S. aureus* data, the contribution of rich medium ingredients to acetate peak integral was accounted by subtracting the spectrum recorded of the medium as a blank. For *E. coli* curves data were fitted to a sigmoidal equation (Figure S2).^22^ In the case of *S. aureus* (Figures S3 and S4), the secretion rate of formate was derived by fitting the NMR time-course data to a sigmoidal function, as done for *E. coli* data. Secretion rate of acetate, not reaching a plateau within 20 h, was derived by a linear fit and the final concentration was estimated at the fixed time interval of 20 h from the start of spectra acquisition.

### Viability Tests

Viability tests were carried out in the presence of different concentrations of kanamycin (KAN), ampicillin (AMP), caprylic acid (CA), irgasan (IRG), graphene-like nanoparticles (GL-NP), and silver nanoparticles (AgNP), (Table S1). All the compounds were purchased from Merck except for caprylic acid (CA) purchased from Carlo Erba (Milan, Italy) and GL-NP which were synthesized in house. IRG-loaded silicone tubes were prepared as previously described.^22^ Samples were prepared by adding the proper amount of compounds to 1 mL of bacterial cell culture in m.m. prepared according to Scheme S1. Cultures were incubated for 24 h at 37 °C, properly diluted, and then dispersed on yeast agar plates for *E. coli* or on BHI agar plates for *S. aureus*, respectively. The agar plates were incubated at 37 °C for 24 h, and the colonies were counted. Results are expressed as the logarithm of CFU/mL considering the dilution necessary to obtain a number of colonies easily countable (up to 400 colonies).

### Cell Integrity at the End of NMR Acquisition Time

To assess cell integrity, spectra acquired at the end of the NMR acquisition period at the plateau phase were compared to spectra recorded on lysed samples. Lysed samples were prepared as follows: cells were harvested by centrifugation, and the cell pellet was washed with potassium phosphate to remove secreted metabolites. The cell pellet was then suspended in 800 μL, and cells were lysed by six cycles of freeze and thaw. The lysate was clarified by centrifugation, and the supernatant was analyzed by NMR. This procedure was applied both to the pellet obtained from samples grown directly in the NMR tube and to a pellet derived from a 10 mL culture grown under identical NMR conditions.

### GL-NP Synthesis

GL NPs were synthesized as reported by Olivi et al.^2^ through a top-down approach using carbon black (CB N110, Sid Richardson Carbon Co., Fort Worth, TX, USA) as a precursor. The process involved grinding the carbon black powder and oxidizing it with concentrated nitric acid (HNO3, 67%) under reflux for 90 h at 100 °C. The oxidized sample was then reduced with hydrazine hydrate (35 μL of hydrazine hydrate per mg of sample) at the same temperature for 24 h. The excess hydrazine was neutralized, and the resulting GL nanoparticles were purified via centrifugation and vacuum filtration. The final product was diluted in distilled water (1 wt %) and stored. Ultrasonic agitation was applied before use to maintain the uniformity of the concentration. Individual GL layers presented vertical sizes ranging from approximately 1 nm to a few nanometers and lateral dimensions of a few tens of nanometers (60–70 nm).^33^

### Scanning Electron Microscopy (SEM)

*E. coli* was grown on site in m.m. alone and in the presence of 0,05 μg/mL AgNP. Subsequently, aliquots of 2 mL were removed and centrifuged at 60000 rpm for 4 min, and the pellet was washed in KPi buffer pH 7.2 and centrifuged again. The pellet was resuspended in KPi, 30 μL was deposited on agar 8%. Samples were fixed with 2,5% glutaraldehyde in saline solution at 4 °C. The samples were rinsed once with a saline solution and further dehydrated using serial diluted ethanol solutions (30, 50, 70, 90, and 100%) for 10 min in each solution. The samples were dried at room temperature and coated with 5 nm of Au by plasma sputtering before imaging. SEM analysis was performed using a Phenom Pro desktop scanning electron microscope (Thermo Fisher Scientific Inc., Eindhoven, The Netherlands), at an accelerating voltage of 15.0 kV, by acquiring the images simultaneously with both backscattered and secondary electron detectors, in mixed mode.

### Statistical Analysis

All data were analyzed using Sigmaplot software (version 12.0 for Windows) and are expressed as the mean ± standard deviation of three independent experiments. Differences between samples treated with antibiotics and control were assessed by one-way analysis of variance (ANOVA) followed by a Bonferroni post hoc test. A 95% confidence level was applied to all statistical analyses, and *p*-values of ≥0.05 were considered not statistically significant. Statistical analysis of all samples treated with antibacterial agent with respect to untreated ones were evaluated using two-tailed Student’s *t* test.

## Results and Discussion

### Real-Time NMR Analysis of *E. coli* Secreted Metabolites in Selected Minimal Media

To enable reproducible NMR-based metabolic profiling and improve spectral readability, a cell growth minimal medium (m.m.) formulation with a defined and controlled chemical composition was employed. This custom medium recipe excluded vitamins to reduce NMR spectral crowding, and CaCl_2_ to not increase bacterial membrane permeability, respectively. The absence of CaCl_2_ was particularly important to avoid potential biases in evaluating antibacterial effects. The ^1^H NMR spectrum of the minimal medium alone showed only the resonances of glucose, which served as the sole carbon source (Figure 1A, a). Spectra were acquired continuously during bacteria growth (Figure S5) and the progressive increase of new signals, assigned to formate, succinate, pyruvate, acetate, lactate, and ethanol, was observed (Figure 1A,b). The increase of the NMR signals over time, exhibiting a characteristic sigmoidal profile reaching a plateau after approximately 20 h (Figure 1B–G), provides evidence of the exometabolite secretion kinetics.

**Figure 1:**
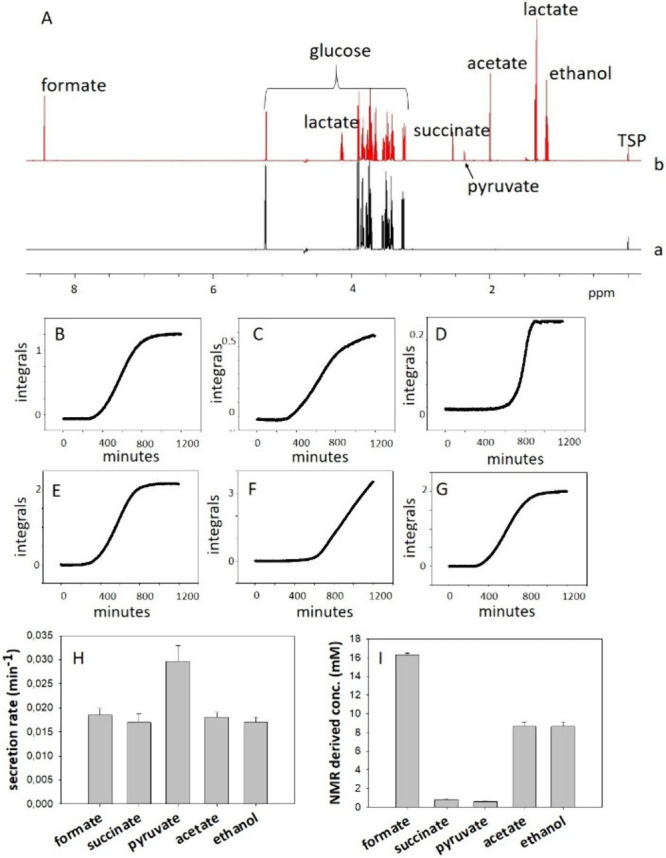
(A) 1D ^1^H NMR experiment acquired on minimal media (a) and on *E. coli* sample after 20 h of glucose fermentation (b). Secreted metabolites assignments are indicated. Proton resonances used as probes to monitor secretion are indicated in Figure S1. (B–G) Secretion curves of *E. coli* exometabolites: formate (B), succinate (C), pyruvate (D), acetate (E), lactate (F), and ethanol (G). (H, I) Secretion rates (H) and end-point concentrations of exometabolites (I). The error bars correspond to the standard deviation of three independent experiments.

The chemical nature of the observed metabolites and the high mM concentrations reached in the supernatant confirm that we are looking at exometabolites, produced and secreted when *E. coli* grows in anaerobic conditions and cells divert the metabolic flow toward fermentation.^34^ A clear shift from aerobic to anaerobic metabolism was evident when comparing spectra acquired at different time-steps along an overnight data recording (Supporting Information and Figure S6). In the spectra recorded at the end-point of the secretion curve, signals of residual glucose (nutrient) are still present (Figure 1A,b). Indeed, when glucose is in excess and oxygen is deficient, the accumulation of acid exometabolites lowers the pH of the medium disfavoring further cell growth and complete glucose consumption.^35–38^

Further evidence that the observed signals are due to secreted metabolites and not to intracellular metabolites released after cell lysis or due to leakage, derive from viability and cell integrity tests performed on samples taken from the NMR tube at the end of NMR acquisition time (see Supporting Information, Figure S7 and S8).

Additional exometabolites, such as putrescine, threonine, valine, citrate, and fumarate, were detected (Figure S9). However, in light of their low concentrations they were not selected as probes to follow the response of bacteria to the presence of antibacterial agents.

The comparison of the secretion curves obtained for most concentrated exometabolites demonstrated that formate, succinate, acetate, and ethanol showed similar lag phases, while pyruvate and lactate exhibited delayed onset of secretion. This delay may be attributed to a slow metabolic shift: as glycolysis slows, Krebs cycle-related intermediates such as acetate and succinate begin to accumulate, followed by an increased release of pyruvate and lactate, which are key molecules in the fermentative pathway. Lactate production increases to regenerate NAD^+^ under anaerobic conditions. All metabolites, except lactate, reached a plateau, indicating a steady-state fermentative condition. Lactate levels continued to increase during the 20 h observation period, suggesting an ongoing shift toward fermentative anaerobic metabolism. By fitting the kinetic curves to a sigmoidal growth model,^22^ secretion rates were calculated (Figure 1H). Formate, acetate, succinate, and ethanol exhibited comparable secretion rates, while pyruvate showed the highest one. Lactate secretion could not be fitted to a sigmoidal model due to the absence of a plateau within 24 h after observation. As shown in Figure 1 I, formate, acetate, and ethanol exhibited the highest end-point concentrations, measured when the secretion curve reached a plateau.

### *E. coli* Exometabolites Secretion Rate, Lag-Phase, End-Point Concentration in the Presence of Antibiotics with Known Mechanism of Action (MoA)

NMR-based metabolomics has previously been used to assess the intracellular and extracellular metabolic responses of *E. coli* to antibiotics with distinct MoAs.^27^ Building on this approach, we investigated whether the kinetic parameters of the secreted metabolites (lag phase, secretion rate, and end-point concentration) could be used to distinguish antibiotics that interfere with intracellular pathways from those that target the cell wall. To this end, we selected two antibiotics with well-characterized and distinct modes of action: kanamycin, which targets the bacterial ribosome and inhibits protein synthesis, and ampicillin, which disrupts peptidoglycan cross-linking in the bacterial cell wall, leading to membrane leakage.^27,39^ Antibiotic concentrations were chosen based on viability assays to ensure cell survival until the end of NMR experiments and comparable cell densities at 24 h, thus minimizing metabolic differences due to disparities in cell growth conditions and density. Specifically, antibiotic concentrations in the bacteriostatic range, that induce a 1 log or 3 log reduction in CFU were selected, namely 0.24 or 2.4 μM for ampicillin and 10 or 19 μM for kanamycin (Figure S10). Under these conditions, both antibiotics induced measurable changes in metabolic secretion profiles compared to untreated *E. coli* (Figure 2A). In particular, the addition of both molecules slowed down secretion rates and prolonged lag phases in a dose dependent way (Figure 2B,D,F). In untreated *E. coli*, the lag phase typically ranged between 3 and 4 h for acetate. The presence of antibiotics prolonged the lag phase to ∼5 h in the presence of ampicillin and up to ∼30 h in the presence of kanamycin (Figure 2A,F).

**Figure 2:**
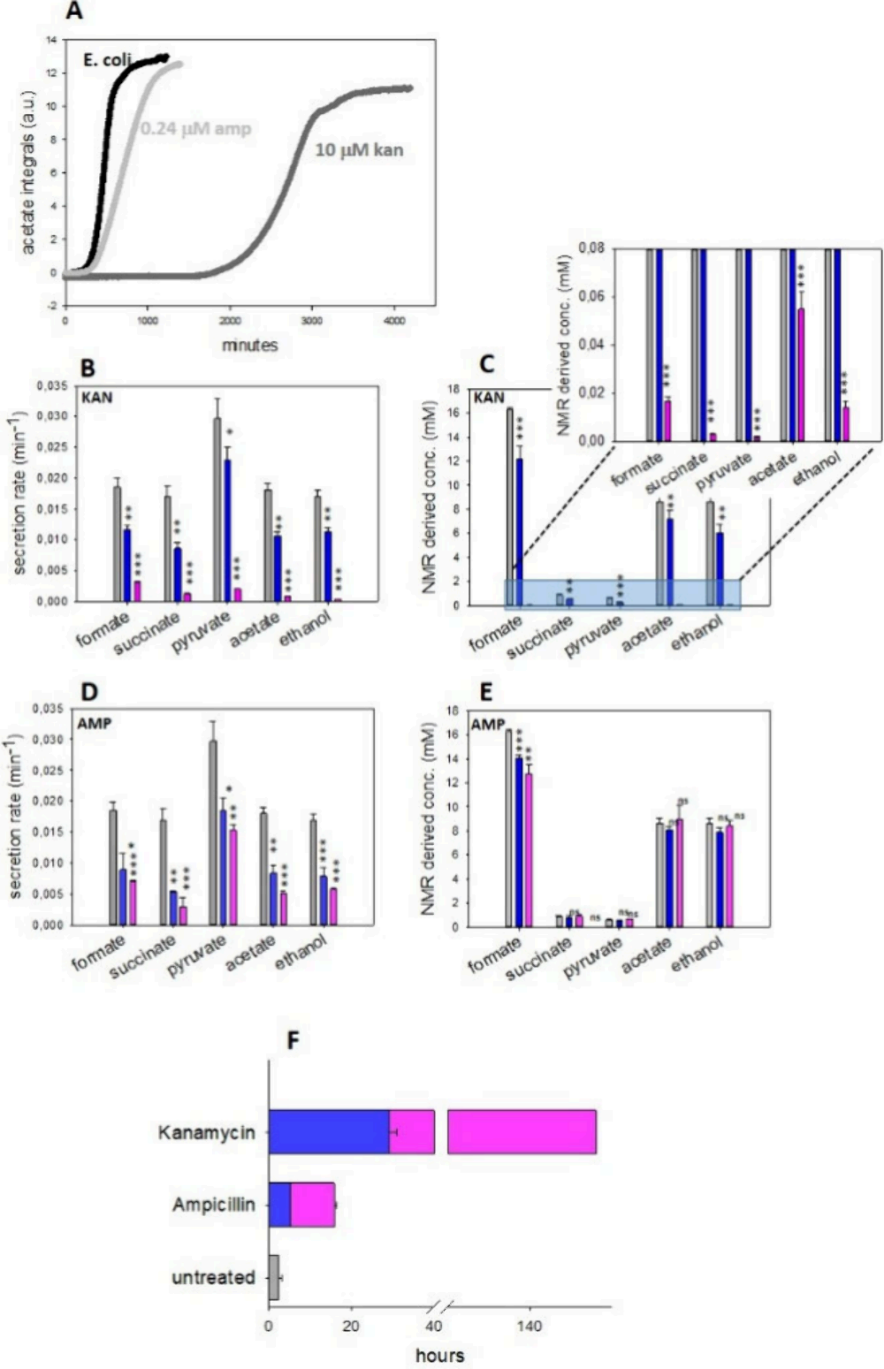
Secretion kinetics of *E. coli* exometabolites. (A) Acetate secretion curves of untreated *E. coli* (black) and in the presence of kanamycin 10 μM (light gray) and ampicillin 0.24 μM (dark gray). Secretion rates (B, D), end point concentrations (C, E), and lag phase duration (F) for untreated *E. coli* (gray) or grown in the presence of kanamycin 10 (blue bars) and 19 (pink bars) μM (A, B), ampicillin 0.24 (blue bars) and 2.4 (pink) μM (C, D). Differences between treated samples and untreated *E. coli* were evaluated using two-tailed Student’s *t* test: ns, *p* > 0.05; *, *p* < 0.05; **, *p* < 0.01; ***, *p* < 0.001.

Interestingly, the metabolite end-point concentrations at the plateau for *E. coli* in the absence and presence of ampicillin are comparable, while they are substantially reduced when bacteria are treated with kanamycin. The measured differences in the end-point concentrations are statistically significant for all metabolites at the highest tested antibiotic concentrations (Figure S11). Differences are not related to a differential loss of cell integrity, as assessed by viability tests performed at the end of the NMR acquisition time (Figure S7). The maintenance of the end-point exometabolites concentrations in the presence of ampicillin correlates well with similar residual glucose (nutrient) concentrations. The conservation of the end-point concentrations is independent of the number of cells contributing to the total amount of secreted metabolites. Although the number of cells is lower in the presence of antibiotics than in their absence (Figure S12), the amounts of secreted metabolites increase significantly with respect to untreated cells, due to stress conditions (Table S3), in line with literature data.^40^

Altogether NMR data indicate that the end-point concentrations of secreted metabolites were strongly reduced by kanamycin, acting by targeting intracellular processes, while they appeared to be preserved in the presence of ampicillin, which is known to perturb the cell membrane. While kanamycin suppresses metabolite production or release, ampicillin slows metabolite export but preserves total metabolite yield.

These results indicate that the specific MoA of antibacterial agents is clearly reflected in the NMR-derived exometabolite secretion kinetics, suggesting the potential use of the derived parameters in distinguishing antibiotics that act on intracellular targets from those that disrupt the bacterial cell envelope.

### Analysis of Secreted Metabolites of *E. coli* Grown in the Presence of Molecules and Functionalized Material with a Known Mechanism of Action

To evaluate whether NMR-derived parameters could be systematically used to define the antibacterial mechanism of action, KINEXO was applied to several agents with a known MoA.

Irgasan (IRG, targeting intracellular processes), caprylic acid (CA, a membrane-disrupting fatty acid), and a functionalized silicon material (T4, acting through surface-mediated contact killing mechanism), differing in chemical/physical nature and mode of action, were investigated. Each agent was tested at bacteriostatic concentrations that yielded log reduction in CFU in the range 1–3.

#### Irgasan

IRG, a broad spectrum antimicrobial agent, exerts its activity by binding to the fabI-enoylacyl carrier protein reductase encoded, thus inhibiting fatty acid biosynthesis.^41^ To evaluate the effect of IRG on the exometabolome of *E. coli*, viability assays were first performed in minimal medium across a range of IRG concentrations. A concentration of 0.0005 mg/L IRG was selected for kinetic analysis as it resulted in a 1-log reduction in viability assays after 24 h (Figure 3A). In the presence of 0.0005 mg/L IRG, *E. coli* secreted the typical set of metabolites (formate, succinate, pyruvate, acetate, lactate, threonine, and ethanol) with secretion rates and end point concentrations at plateau markedly reduced compared to controls untreated (Figure 3B,C). This reduction was particularly pronounced for formate, succinate, and acetate. The kinetics features observed in the presence of IRG closely resemble those observed in the presence of kanamycin, which is consistent with IRG targeting an intracellular enzyme and its bacteriostatic mode of action. At a higher IRG concentration (0.001 mg/L), the NMR spectra revealed only weak signals corresponding to acetate and lactate (Figure 3 B,C), indicating a further suppression of metabolic activity. Ethanol, although produced, could not be quantified because of its dual origin: it is secreted by *E. coli* and used as a solvent for IRG, affecting signal integration. Similarly, lactate, which did not reach a plateau in 20 h under standard conditions, was excluded from the comparative analysis. Importantly, no significant pH change was observed after 20 h of growth in the presence of 0.0005 mg/L IRG compared to untreated cultures, where the pH dropped from 7.0 to ∼4.5 due to the accumulation of acidic metabolites. Similarly to what was observed for antibiotics, the lag phase was prolonged as a function of IRG concentration: in the presence of 0.0005 mg/L IRG, the lag phase of acetate extended to approximately 24 h and increased to multiple days at 0.001 mg/L.

**Figure 3:**
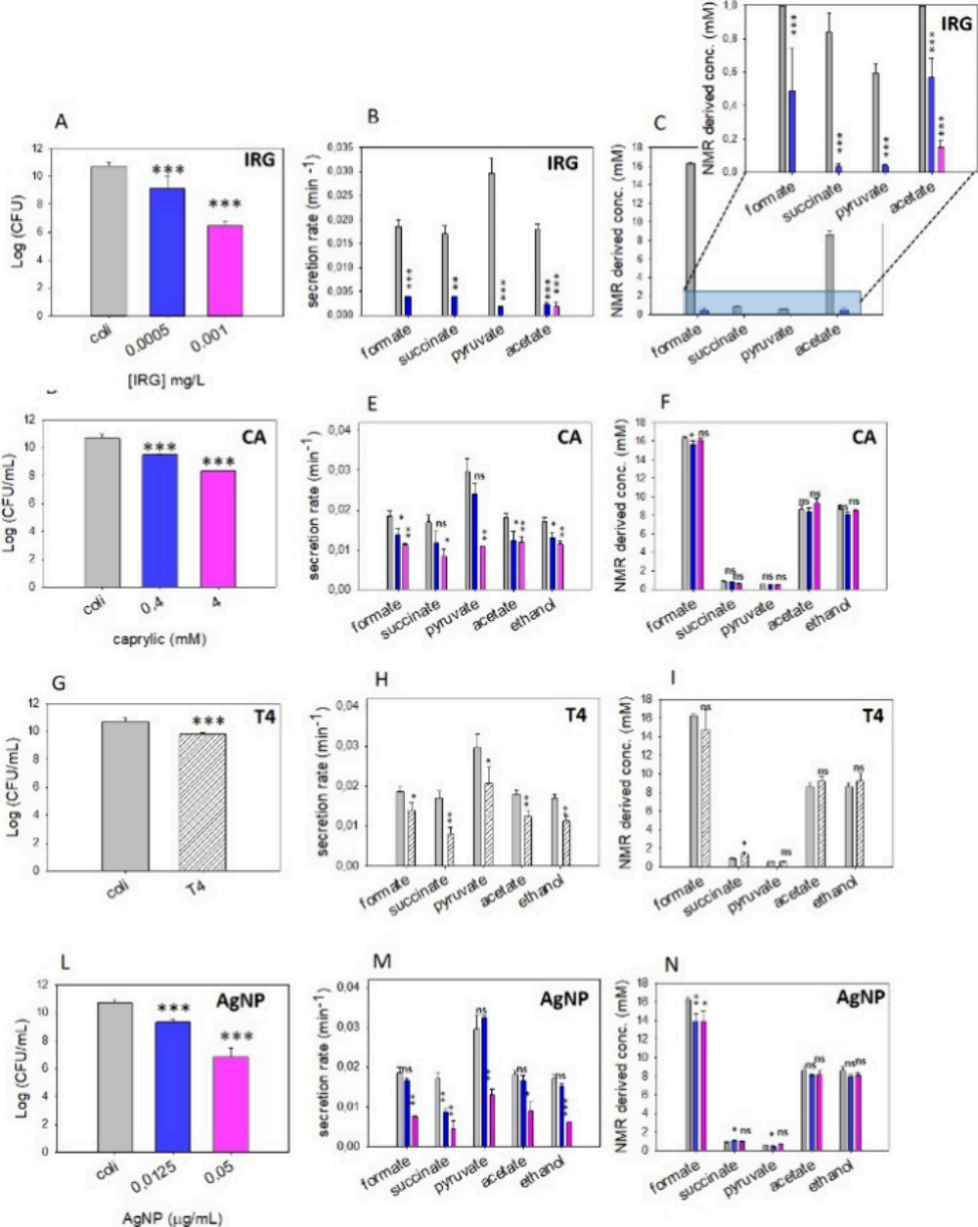
Viability tests, metabolite secretion rates, and end point concentrations observed for *E. coli* in the absence (gray bars) and presence of (A–C) 0.0005 (blue) and 0.001 (pink) mg/L of IRG (blue), (D–F) 0.4 (blue) and 4 (pink) mM CA, (G–I) T4 silicon tubes (stripped), (L–N) 0.0125 (blue) and 0.05 (pink) μg/mL AgNP. Data are presented as the mean ± standard deviation. Differences between treated and untreated *E. coli* samples were evaluated using two-tailed Student’s *t* test; ns, *p* > 0.05; **, *p* < 0.01; ***, *p* < 0.001.

Altogether, NMR data suggest that IRG significantly impairs glucose metabolism, which is consistent with its known inhibitory effects on fatty acid synthesis and reported disruptions in glycolytic and TCA cycle regulation.^42^ Taken together, these results reinforce the correlation between intracellular-acting drugs and strong reductions in both metabolite secretion rates, end-point concentrations, and secretion lag phase lengthening, as observed for kanamycin.

#### Caprylic Acid

Fatty acids have gained increasing attention as promising next-generation antimicrobial agents due to their ability to disrupt bacterial cell membranes.^43^ Among them, caprylic acid (CA), a natural medium chain fatty acid (C8), exhibits broad-spectrum antibacterial activity and is widely used in the food industry.^44,45^ Caprylic acid has also found application in the preparation of flexible bactericidal polyurethane foams based on soybean and linseed oil polyols.^46,47^ To assess the effect of CA on *E. coli* metabolism, viability assays were performed at a variety of CA concentrations. Concentrations of 0.4 and 4 mM, which induced a 1 and 3 log reduction in CFU after 24 h respectively, were selected for kinetic NMR analysis (Figure 3D). In the presence of CA, *E. coli* retained the ability to secrete metabolites, such as formate, acetate, succinate, pyruvate, lactate, and ethanol. Secretion rates of formate, acetate, and ethanol were particularly reduced in the presence of 0.4 mM CA (Figure 3E), while all metabolites secretion rates were significantly reduced in the presence of 4 mM CA. Lag phases were elongated in a dose dependent manner. Interestingly, despite slower metabolic activity, the end point concentrations of exometabolites remained comparable to those observed in the absence of CA at the two tested concentrations (Figure 3F). The secretion kinetics profile, characterized by delayed onset of secretion and preserved final metabolite levels, mirrored the metabolic phenotype observed in the presence of ampicillin, which disrupts the bacterial cell wall and causes leakage while preserving some metabolic functions.^43^ These findings are consistent with the proposed MoA of CA related to perturbations of membrane integrity^43^ rather than interference with intracellular pathways.

#### Silicon Material Loaded with IRG

As a further proof of concept, we applied the proposed NMR-based metabolite kinetic profiling method to a silicon-based material functionalized with IRG (referred to as T4). This material has previously been shown to exert antibacterial activity against both *E. coli* and *S. aureus*, primarily through a surface-mediated contact killing mechanism with minimal release of the active compound.^22^ To assess whether the exometabolites secretion kinetics parameters could capture the mode of action of this material, viability assays and real-time NMR monitoring were conducted in the presence of T4. Silicon samples were deposited at the bottom of the NMR tube so that they did not fluctuate during acquisition. As shown in Figure 3H,I, the metabolic response of *E. coli* in the presence of T4 mirrored the profiles observed for ampicillin and caprylic acid. Specifically, a consistent decrease in secretion rates was observed for all major metabolites, while end point concentrations at the plateau remained comparable to untreated controls (Figure 3H,I). Exometabolites secretion lag phases were elongated with respect to untreated *E. coli*. These results are in line with our previous findings that T4 exerts a bactericidal effect through physical interaction with the bacterial membrane rather than interfering with intracellular targets.^22^ The observed slowdown in exometabolite secretion rates is consistent with membrane stress-induced growth delay, while the unaffected metabolite plateau concentrations indicate that sugar metabolism was not affected. Thus, KINEXO supports a contact-dependent, cell-envelope-disrupting MoA. These data further validate the ability of NMR-based exometabolites kinetics to differentiate antimicrobial agents according to their MoA.

### NMR-Based Exometabolites Secretion Parameters Cluster Defining Antibacterial MoAs

The discriminative power of exometabolites secretion parameters is highlighted in 3D plots generated using secretion lag phase, rate, and end-point concentration of exometabolites at plateau. Interestingly, these parameters clustered in two distinct groups with agents disrupting the cell wall or membrane (ampicillin, CA, and T4) gathering together (Figure 4, green circle), while agents targeting intracellular biosynthetic pathways (kanamycin, IRG) form a separate cluster (Figure 4, blue circle). The duration of the secretion lag phase appears particularly sensitive in distinguishing agents that act on intracellular targets from those that affect the bacterial membrane. While intracellular targeting agents induced delays of up to 24 h or more, membrane targeting agents caused only modest lag phase extensions relative to untreated controls. These findings support the use of KINEXO as a robust and rapid approach to infer the MoA of antibacterial compounds.

**Figure 4:**
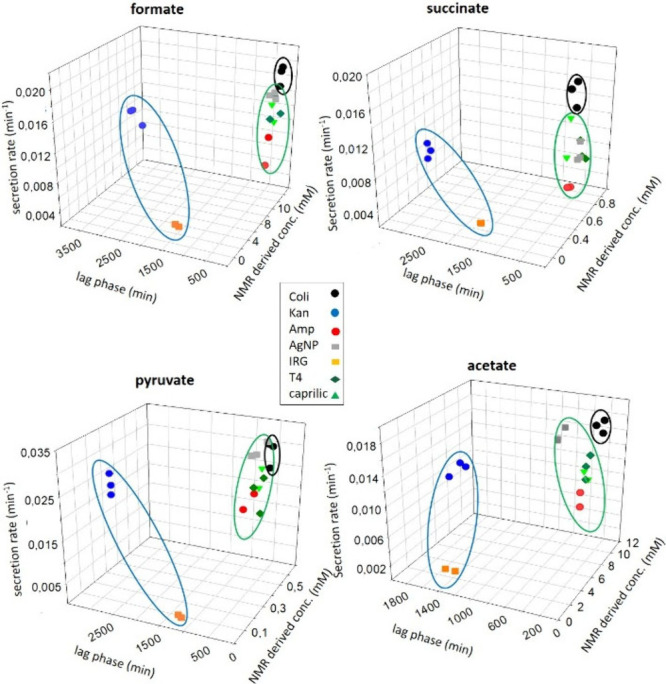
3D graphs reporting secretion lag phase, secretion rates, and end point concentrations at the plateau for *E. coli* in the absence and in the presence of kanamycin 10 μM, ampicillin 0.24 μM, 0.0125 μg/mL AgNP, 0.4 mM CA, 0.0005 mg/L IRG and T4, yielding 1 log reduction in CFU. Three independent measurements recorded in three different samples were considered for each antibacterial agent. Clusters are highlighted with green and blue circles.

#### 3D Plot Analysis To Propose the AgNP Mechanism of Action

To further test KINEXO, we investigated the MoA of silver nanoparticles through a combination of NMR analysis and scanning electron microscopy (SEM). AgNPs are known for their potent antimicrobial properties, although their exact MoA remains debated. Evidence suggests that their effects are multifaceted, with membrane interaction being a dominant mechanism.^48^ AgNPs have been shown to physically associate with bacterial membranes, especially in the case of Gram-negative bacteria such as *E. coli*, where the electrostatic attraction between negatively charged lipopolysaccharides and positively charged silver ions facilitates membrane penetration and structural disruption.^49,50^ Smaller nanoparticles (<10 nm) exhibit enhanced antibacterial activity due to increased surface area and reactivity,^48^ and it was reported that they attach to the surface of the cell membrane and drastically affect permeability and respiration. Small AgNPs have also been reported to be able to penetrate bacteria and cause further damage.^51^ Generally, the antimicrobial activity of AgNPs measuring 20–80 nm has been mainly attributed to the release of silver ions.^52^ We investigated the exometabolites of *E. coli* samples exposed to commercial AgNPs with a nominal diameter of 10 nm, lying at the borderline of the two classes of NPs, raising the possibility of dual or overlapping modes of action. Viability assays were performed to select effective concentrations of AgNPs and those that reduce the *E. coli* cell viability by 1 and 3 log CFU (Figure 3L, Figure S13). At the two concentrations selected, AgNPs induced a slowdown of the metabolite secretion, especially in the presence of 0.05 μg/mL AgNP, while plateau concentrations of most metabolites (except formate) were still reached, albeit at a longer time lapse (Figure 3M,N). When the duration of the lag phase, the secretion rate, and the concentration of the metabolite at the plateau were plotted in the 3D graph (Figure 4), AgNP-treated samples clustered with agents known to interfere with the cell wall (e.g., ampicillin, CA and T4) (green circle). These results suggest that, despite their borderline size, AgNPs at the tested concentrations exert their antibacterial activity primarily via interactions with the bacterial cell envelope. SEM analysis of *E. coli* cells treated with 0.05 μg/mL AgNP for 24 h (Figure S14) showed that cells exposed to AgNP displayed clear morphological alterations compared to untreated controls, including surface disruption and membrane collapse, consistent with prior literature reports.^53–55^

Taken together, NMR and SEM analyses converge on a membrane-damaging effect as the dominant MoA for 10 nm AgNPs.

### Analysis of *S. aureus* Secreted Metabolites in the Presence of Antibacterial Agents

Almost all microorganisms secrete metabolites;^31^ thus, in principle, the NMR-based analysis of exometabolites could be broadly applied to investigate the MoA of molecules and materials in a variety of bacterial species. As a proof of concept, we tested whether the proposed approach could be easily extended to a Gram-positive bacterium, *Staphylococcus aureus*. To this end, we optimized the cell culture conditions to cultivate *S. aureus* in minimal medium to ensure reproducible growth and sufficient metabolite production for NMR detection (see the Experimental Section). We then evaluated the effects on the *S. aureus* exometabolome of three antimicrobial agents: Graphene-like nanoparticles (GL-NP) and IRG, which have a known mechanism of action, together with 10 nm commercial silver nanoparticles (AgNPs), whose mechanism of action has not been fully described. Among the detected metabolites, formate was selected as the primary probe for kinetic analysis due to its well-resolved NMR signal, rapid accumulation in 20 h at 37 °C, and limited involvement in downstream metabolism. Acetate was also considered as a probe, and its kinetic parameters were derived, as discussed in the experimental section.

#### Graphene-like Nanoparticles

It was demonstrated by Olivi and co-workers^2^ that graphene-like nanoparticles (GL NPs), derived from carbon black by chemical demolition, exhibit antibacterial activity against *S. aureus* in a concentration-dependent manner after 24 h of exposure and have demonstrated biocompatibility in vertebrate models.^33^ Biological studies in murine fibroblasts, human keratinocytes, and HeLa cells^56^ confirmed no cytotoxic effects or disruptions in key biological parameters. According to previous findings, bacterial viability was reduced by approximately 96% at 50 μg/L GL NPs, compared to untreated controls.^2^ In our study, the effect of GL-NPs on *S. aureus* was evaluated through viability and NMR tests at 10, 50, and 100 μg/L GL NP (Figure 5A–C). The formate secretion rate was found to decrease progressively with increasing GL-NP concentrations, with the most marked reduction observed at 100 μg/L. Despite the slower secretion rate, the final concentrations of formate and acetate remained relatively constant, within the experimental error, at all tested concentrations (Table S4). These results suggest that while cell growth and metabolism are delayed, bacteria ultimately reach a comparable level of metabolic activity in terms of extracellular formate and acetate accumulation, reflecting the behavior observed in *E. coli* exposed to membrane-targeting agents such as caprylic acid and ampicillin. The NMR-derived kinetic data for *S. aureus* in the presence of GL NPs are consistent with a MoA that primarily involves cell envelope disruption. This interpretation aligns with the hypothesis proposed by Olivi et al.,^2^ who suggested that GL NPs exert their antibacterial effect through a coating or wrapping mechanism, which leads to mechanical damage to the bacterial membrane. Together, the results reinforce the utility of real-time NMR analysis of secreted metabolites for distinguishing bactericidal agents targeting the cell wall from those targeting intracellular processes, demonstrating the broader applicability of the method to different bacterial species and nanomaterials.

**Figure 5:**
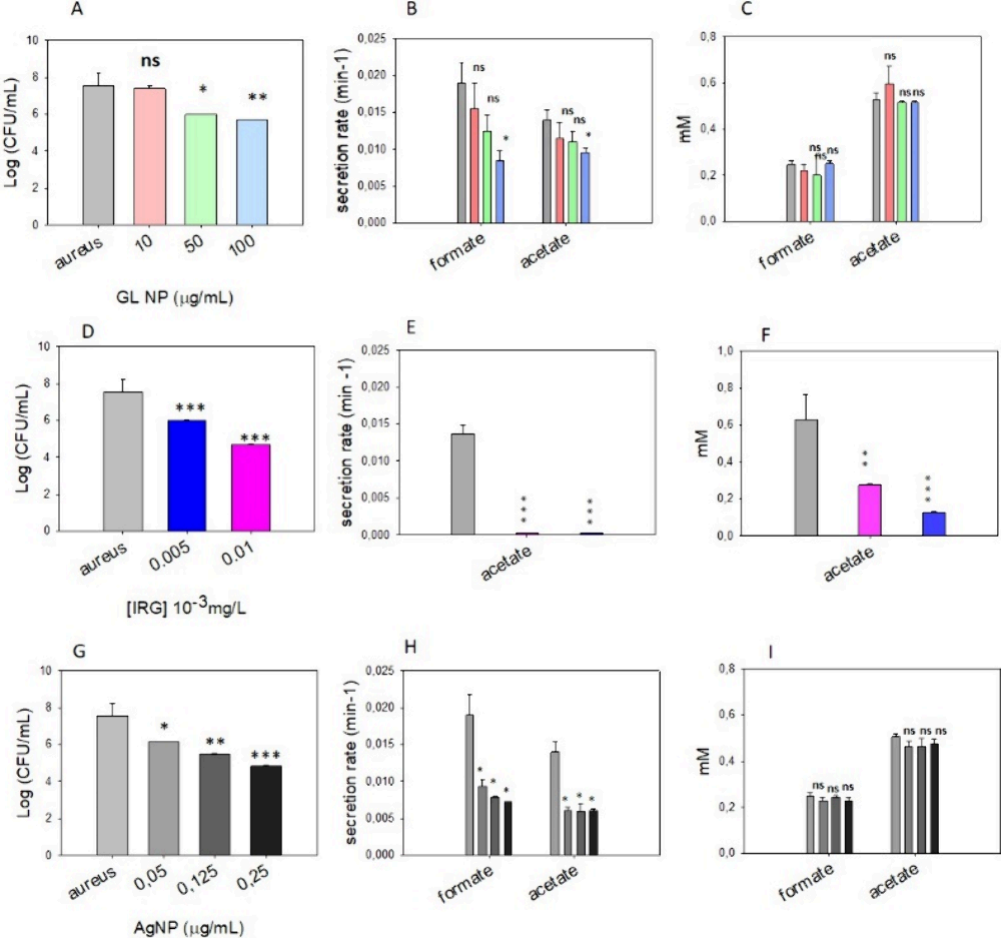
*S. aureus* viability test, formate and acetate secretion rates, and plateau concentration at 20 h after inoculation as observed for bacteria grown in the absence (light gray) and presence of 10, 50, and 100 μg/L GL NP (A–C); 0.005 × 10^–3^ (blue) and 0.01 × 10^–3^ (pink) mg/L of IRG (D–F); 0.05, 0.125, and 0.25 μg/mL of AgNP (G–I). Experiments were performed in triplicate. Data are presented as the mean ± standard deviation. Differences between treated samples and untreated *S. aureus* were evaluated using two-tailed Student’s *t* test: ns, *p* > 0.05; **, *p* < 0.01; ***, *p* < 0.001.

#### Irgasan

As discussed above, Irgasan (IRG) is effective against a broad range of bacterial species, including *S. aureus*, through a well-characterized intracellular mechanism targeting fatty acid biosynthesis. Viability tests (Figure 5D) confirmed a dose-dependent reduction in *S. aureus* CFU counts after 24 h of exposure to 0.005 and 0.01 × 10^–3^ mg/L of IRG. In the NMR spectra collected from these samples, formate was not detected at either concentrations, even at extended acquisition times, indicating their suppression of its production or concentrations below the NMR detection threshold. Although small quantities of succinate, pyruvate and aromatic/aliphatic amino acids (likely tyrosine or alanine) were occasionally observed, similar to the findings by Dorries et al.,^57^ the signal intensity was not sufficient for reliable quantification or kinetic analysis. Furthermore, these metabolites were not consistently detected in untreated controls, making direct comparisons inconclusive. Given its abundance and consistent presence, acetate was selected for quantitative analysis. As shown in Figure 5E,F, both the secretion rate and the acetate concentration at 20 h were significantly reduced in the presence of IRG (Table S4). These results are consistent with the bacteriostatic or bactericidal effects of IRG through the inhibition of fatty acid synthesis, specifically inhibiting the FabI enzyme, which appears to universally alter central metabolic activity and metabolite secretion in Gram negative and Gram positive bacterial species.

### 3D Plot Analysis To Highlight the Mechanism of Action of the Antibacterial Agent

To validate the use of NMR-derived exometabolites kinetics for the identification of antibacterial mechanisms in the case of *S. aureus*, the acetate secretion rate and lag phase together with the concentration of acetate at 20 h of cell growth in the absence and presence of bactericidal agents were compared on a 3D plot (Figure 6). The concentrations of bactericidal agents inducing a reduction of 3 log in CFU were selected, allowing a direct comparison of the metabolic responses of bacteria under similar viability conditions. As previously observed for *E. coli*, the metabolic parameters observed in the presence of GL-NP, which exert a mechanical and membrane disruptive effect, were clearly different from those observed in the presence of IRG, which interferes with essential intracellular metabolic pathways (Figure 6). Interestingly, unlike that for *E. coli*, the metabolite lag phase of secretion was only marginally affected by the presence of the different agents in *S. aureus*. These results suggest that lag phase extension may not be as discriminative for Gram-positive bacteria as it is for Gram-negative bacteria, possibly due to intrinsic physiological differences in cell growth dynamics and/or stress responses.

**Figure 6:**
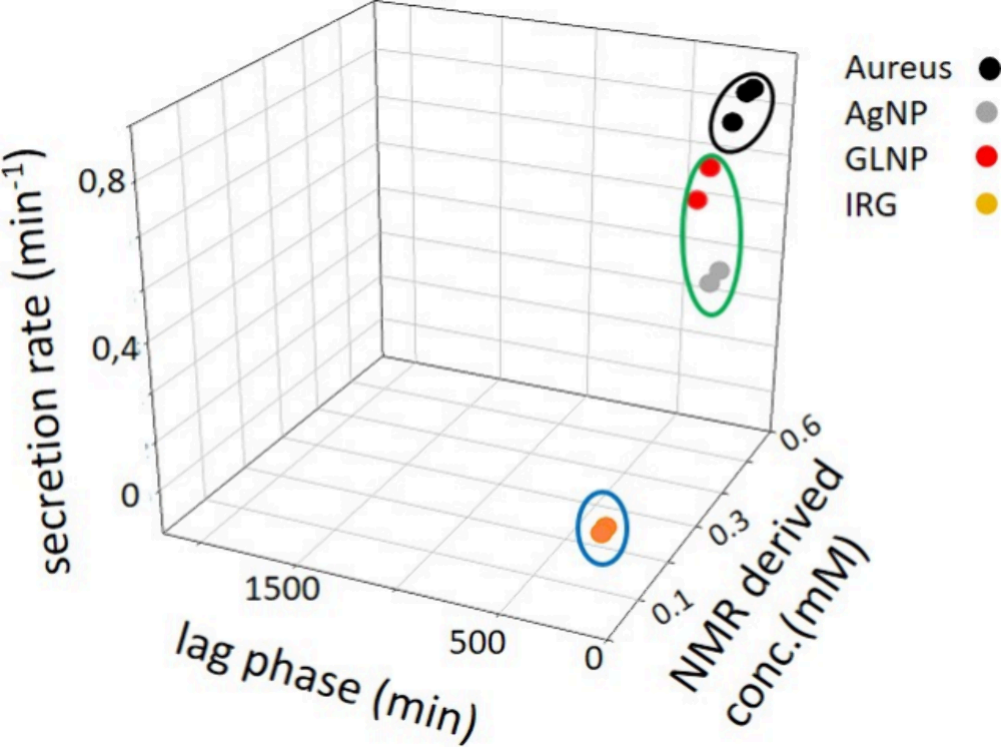
3D plots reporting acetate concentration at 20 h from the inoculation, lag phase duration, and secretion rates for *S. aureus* in the absence and presence of 100 μg/L GL-NP, 0.05 μg/mL Ag NP, and 0.005 × 10^–3^ mg/L IRG. Data measured for untreated *S. aureus* are reported in black.

#### AgNPs

The analysis was then extended to 10 nm AgNPs. Viability tests showed a reduction of 3 log_10_ at 0.05 μg/mL (Figure 5G). The lower sensitivity to AgNP observed for *S. aureus* with respect to *E. coli* is consistent with the reports in the literature and is attributed to the thicker peptidoglycan layer and distinct membrane charge characteristics of Gram-positive bacteria.^49^ Metabolite kinetics was monitored using formate and acetate. In both cases, the secretion rate decreased as the AgNP concentration increased, confirming a dose-dependent suppression of metabolic activity. Kinetic data from cultures exposed to 0.05 μg/mL AgNP grouped in the 3D metabolite parameter spaces together with GL-NP treated samples (Figure 6, green circle). These results support the hypothesis that the AgNPs antibacterial mechanism involves a disruption of the cell wall in *S. aureus*, as previously observed for *E. coli*.

The consistency of clustering patterns observed for Gram-negative and Gram-positive bacteria treated with classes of agents with similar MoA suggests that this approach may be broadly applicable across diverse microbial species.

## Conclusions

In this study, we established a novel, label-free, sample manipulation-free, NMR-based method, KINEXO, to investigate the MoA of antibacterial agents by analyzing the three parameters (secretion lag phase, secretion rate, and metabolites concentration at the plateau) extracted from time-resolved 1D ^1^H NMR analysis of exometabolome. Real-time NMR has already proven value as a monitoring tool and to time-stamp the onset of antibacterial action. The novelty of KINEXO approach is to provide a robust criterion to enable MoA classification by a simple analysis of the exometabolites secretion kinetics. The approach was tested in *Escherichia coli* and *Staphylococcus aureus* as model systems of Gram-negative and Gram-positive bacteria, respectively. The optimization of the experimental conditions, in terms of bacterial growth media and dilution scheme, is crucial and should be fine-tuned for each bacterial strain. Chemically defined minimal media are necessary to reduce the crowding of NMR signals and to finely control secreted metabolic products. It should be noted that the use of minimal growth medium, representing a further source of mild stress, increases the sensitivity of bacteria to external stresses, favoring the MoA investigation. Viability tests are needed to select the ideal concentrations of antibacterial agents for comparative analysis. In this sense, antibacterial concentrations in the bacteriostatic range, which decrease bacteria viability by 1 −3 log_10_ are eligible to obtain good test sensitivity. When new agents are tested, at least two concentrations are needed to assess dose-dependent effects. One of the advantages of KINEXO approach is the real-time measurement, without any need of sample manipulation nor withdrawals, thus substantially reducing bias or error sources. Furthermore, the proposed analytical method does not require isotope labeling and is based on the recording of simple ^1^H 1D NMR experiments that can be easily set up. Our results show that different classes of antibacterial agents induce distinct and characteristic alterations in the secretion kinetics of key exometabolites such as acetate, formate, pyruvate, lactate, and ethanol. In particular, agents affecting the cell wall or membrane integrity led to a reduction in secretion rate, a moderate extension of the lag phase (a few hours), and preserved the final end-point concentrations of metabolites. On the contrary, agents targeting intracellular pathways induced a substantial elongation of the secretion lag phase and a reduction in both the secretion rate and the end-point metabolite concentrations. NMR-derived parameters (secretion lag phase duration, secretion rate, and end-point metabolite concentration) visualized in a 3D plot clearly cluster the data according to MoA of the antibacterial agent. The three parameters therefore form a mechanistically interpretable triad that discriminates intracellular-targeting agents from membrane/envelope disruptors in a single, unified workflow. This explicitly advances beyond prior end point metabolomics approaches generally used for MoA inference, by making kinetics the primary analytical discriminator.

It is worth mentioning that this method is independent of the chemical and physical characteristics of the antibacterial agent to be analyzed. Indeed, the observed parameters are related to the secreted metabolites rather than to the bactericidal agent nature, opening the way for the investigation of nanomaterials and/or materials interacting with bacteria. Given the growing interest in alternative antimicrobials to overcome resistance to bacteria, this approach could serve as a valuable platform for the early stage screening and mechanistic characterization of new antibiotics and antibacterial candidates in general.

## Supplementary Material



## Notes

Chemical structures of the detected secreted metabolites; description of dilution scheme; secretion curves observed for *E. coli*; selection of minimal medium for *S. aureus*; 1D ^1^H NMR of *S. aureus* exometabolites; secretion curves observed for *S. aureus*; series of 1D ^1^H NMR spectra acquired on *E. coli* collected over 20 h; description of aerobic to anaerobic shift; 1D ^1^H NMR spectra acquired on aerobic and anaerobic culture; viability test acquired on NMR samples at the end of acquisition time; NMR-based cell integrity test; 1D ^1^H NMR spectrum recorded after 20 h of *E. coli* fermentation; *E. coli* viability test in the presence of kanamycin and ampicillin; secretion rates and end point concentrations for *E. coli* in the presence of different amounts of antibiotics; growth curve of *E. coli* in the presence of antibiotics; viability test on *E. coli* in the presence of AgNPs; SEM images of *E. coli* alone or incubated with AgNPs; tables listing atibacterial agents and concentrations, *E. coli* exometabolites end point concentration, estimate of secreted metabolite moles per single cell for untreated and treated *E. coli*, and *S. aureus* exometabolites concentration (DOCX)
