# Supplementary material for: NMR Based Real-Time Analysis of Exometabolites Decodes the Mechanism of Action of Antibacterial Molecules, Nanoparticles, and Materials
Source: Anal Chem. 2026 Feb 23;98(9):6772–82. doi: 10.1021/acs.analchem.5c06983 (PMC12980489; doi:10.1021/acs.analchem.5c06983)
Supplement: Supplementary file 1 [file ac5c06983_si_001.docx]

Supporting Information

NMR based real-time analysis of exometabolites decodes the mechanism of action of antibacterial molecules, nanoparticles and materials

Simona Tomaselli *^1^, Roberto K. Salinas ^2^, Michela Alfè ^3^, Valentina Gargiulo ^3^, Simona Losio ^1^, Laura Ragona ^1^

^1^Istituto di Scienze e Tecnologie Chimiche (SCITEC), CNR, via A. Corti 12, 20133 Milan, Italy; ^2^Institute of Chemistry, University of São Paulo, Av. Prof. Lineu Prestes, 748 , 05508-000 São Paulo, Brazil; ^3^Istituto di scienze e tecnologie per l’energia e la mobilità sostenibili (STEMS), CNR,via G. Marconi 4, 80125 Naples, Italy

**TABLE of CONTENTS**

Chemical structures of the detected secreted metabolites; Description of dilution scheme; Secretion curves observed for E. coli; Selection of minimal medium for S. aureus; 1D 1H NMR of S. aureus exometabolites; Secretion curves observed for S. aureus; Series of 1D 1H NMR spectra acquired on E. Coli collected over 20 hours; Description of aerobic to anaerobic shift; 1D 1H NMR spectra acquired on aerobic and anaerobic culture; Viability test acquired on NMR samples at the end of acquisition time; NMR-based cell integrity test; 1D 1H NMR spectrum recorded after 20 hours of E. coli fermentation; E. coli viability test in the presence of kanamycin and ampicillin; Secretion rates and end point concentrations for E. coli in presence of different amount of antibiotics; Growth curve of E. coli in presence of antibiotics.; Vi-ability test on E. coli in the presence of AgNPs; SEM images of E. coli alone or incubated with AgNPs.

Tables: Antibacterial agents and concentrations; E. coli exo-metabolites end point concentration; Estimate of secreted metabolite moles per single cell for untreated and treated E. coli; S. aureus exometabolites concentration.


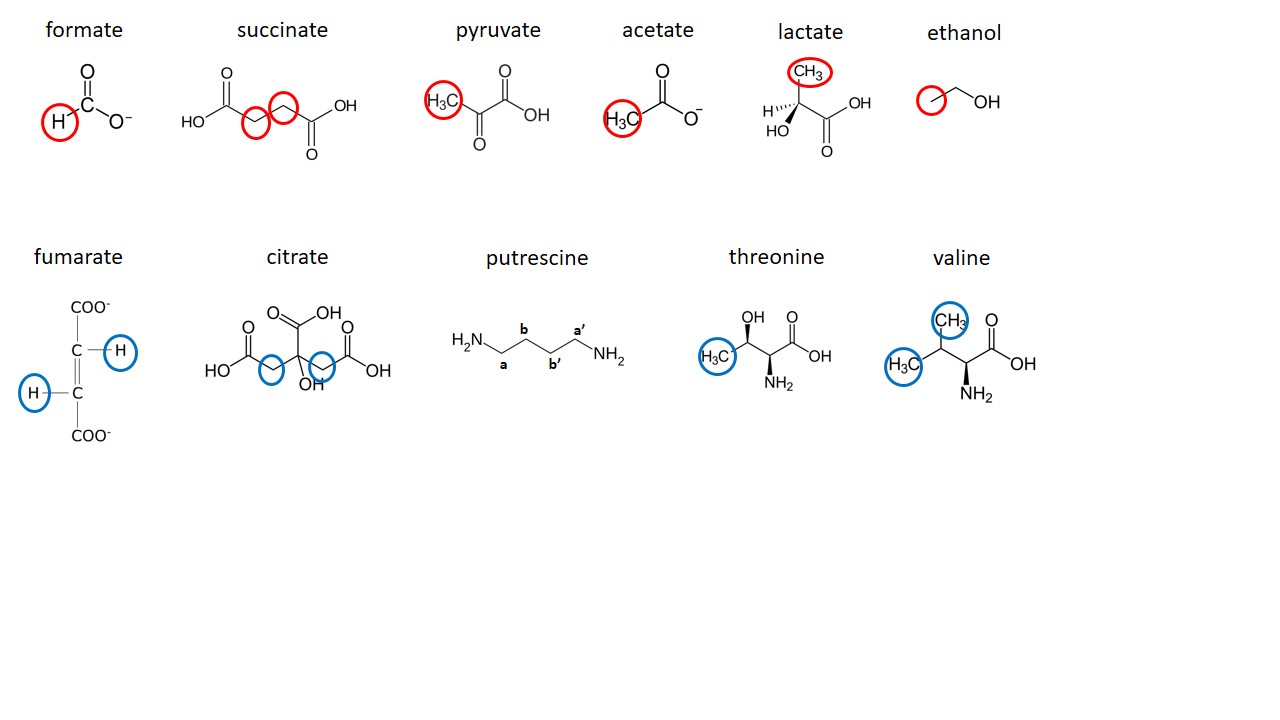


**Figure S1**. Chemical structures of the detected secreted metabolites. Proton employed as probes to study secretion kinetics of the most abundant metabolites (first line) are highlighted in red. Less abundant secreted metabolites (second line) were identified by monitoring protons highlighted in blue. Putrescine protons are labelled.

**Selection of dilution scheme**

In order to set-up the growth/dilution scheme, 18 or 24 hours preculture (Scheme S1 Step B), secreted metabolites kinetic parameters obtained from cells pre-cultured for 18 and 24 hours were compared.


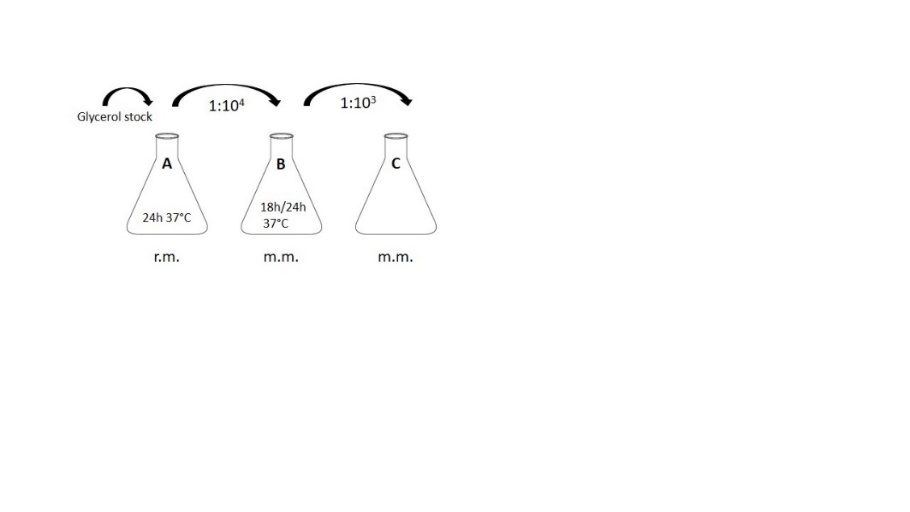


**Scheme S1**. Dilution scheme used to test stress factors.

As an example, Figure S2 A-C compares the kinetic curves of formate, acetate and ethanol registered from cell cultures that were adapted to m.m. for 18 hours (black line) and for 24 hours (grey line). Metabolites secreted by bacteria incubated for 18 hours in m.m. after the first dilution step (Scheme S1 Step B), reached the plateau faster than those cultured longer even though they showed the same metabolites concentration after 20 hours. Figure S2 D compares the computed secretion rates of formate, acetate and ethanol from cell cultures that were adapted for 18 hours (grey bars) or 24 hours (dark red bars) in m.m. (Scheme S1 Step B). For experimental timing reasons all the subsequent experiments were carried out with cultures adapted to m.m. for 18 hours at 37°C (Step B of Scheme S1).

**
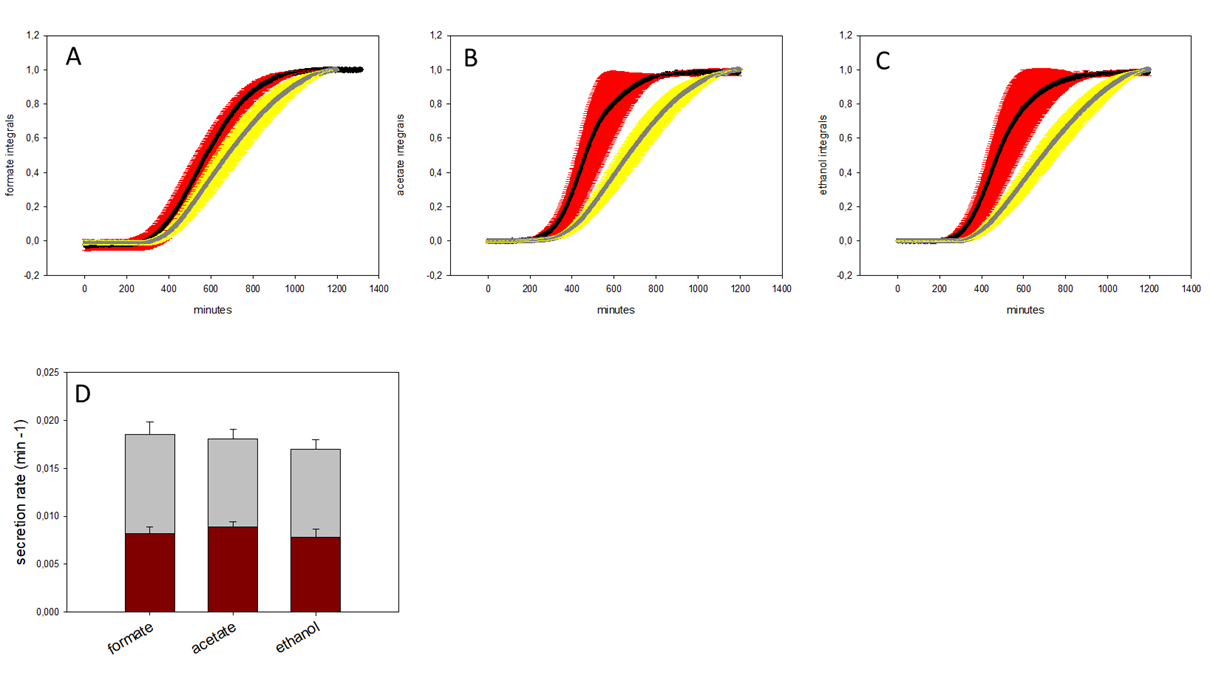
**

**Figure S2**. Secretion curves of formate (A), acetate (B) and ethanol (C) of E. coli growing at 37°C. Data are acquired after incubation of bacteria in m.m. for 18 hours (black curve and red error bars) or 24 hours (grey curve and yellow error bars) after the first dilution step (Scheme 1 in Material and Methods, step B). Reported curves are the mean of three measurements and error is calculated as standard deviation. Data are normalized on the highest integral value obtained at the plateau of each curve. Secretion rates comparison (D) of E. coli grown in m.m. for 18 hours (grey) and 24 hours (dark red) after the first dilution (Scheme 1, step B).

**Selection of minimal medium for S. aureus**

For the purpose of this study diluted rich media with different ratios of potassium phosphate buffer/BHI were explored to balance the NMR (poor or low intense initial 1H signals in order to appreciate intensity changes of secreted metabolites) and growing needs (sufficient nutrients concentration to ensure a reasonable cell growth which means appreciable secreted metabolites concentration variation over a reduced time growth, <24h to reach the plateau).

Under these conditions S. aureus produced and secreted different metabolites, namely formate, acetate, lactate and ethanol as indicated in Figure S3. In Figure S4 are reported the intensity variation curves of secreted metabolites NMR signals as a function of time at two KPi/BHI v/v ratios: 5% (red error bars) and 10% (yellow error bars) BHI. As in the case of E. coli, the kinetic curves showed an initial lag phase (about 3 hours) followed by a growing phase and a plateau. Formate, ethanol and lactate reached a plateau within 20 hours at 37°C, which is a reasonable acquisition time to appreciate changes in terms of curve slope (secretion rate) and plateau (maximum concentration of metabolites). Acetate did not reach a plateau after 20 hours, even when higher concentrations of BHI were considered. Lactate levels decrease after reaching the maximum concentration at 11 hours (~660 min) and 7.5 hours (~450 min) for 5 and 10% respectively. This behavior is however expected since it was demonstrated that S. aureus metabolize lactate producing acetate ^1^. No significant differences were detected in the two different diluted rich media. Hence, 5% BHI semi-rich medium was chosen for the NMR analysis. Further analyses to select a best minimal medium to grow S. aureus are ongoing.


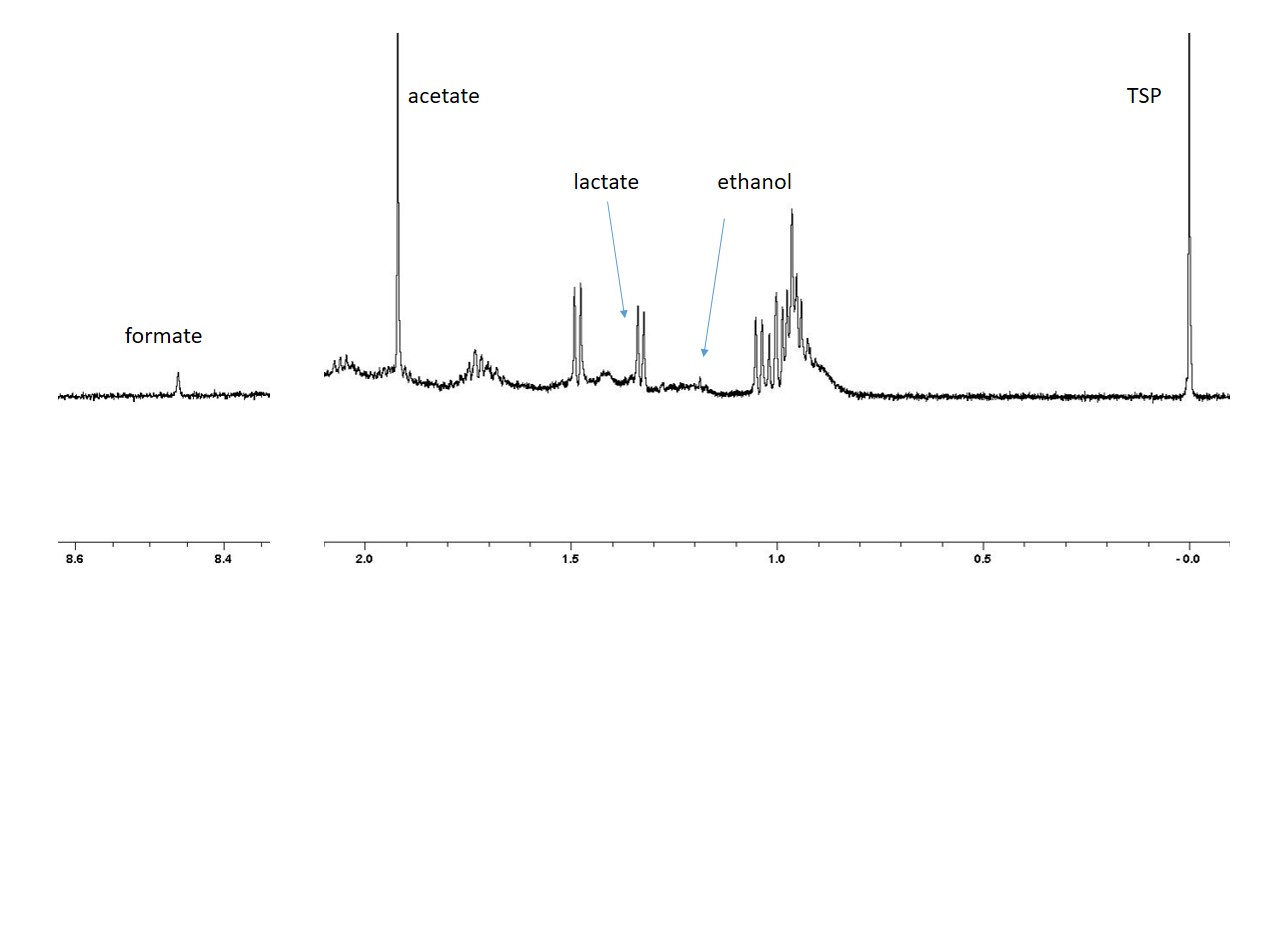


**Figure S3**. 1D 1H NMR experiments acquired after 20 hours of S. aureus fermentation activity in KPi/BHI buffer. The assignments of the NMR signals of exo- metabolites are reported. All other visible resonances were attributed to BHI medium.

**
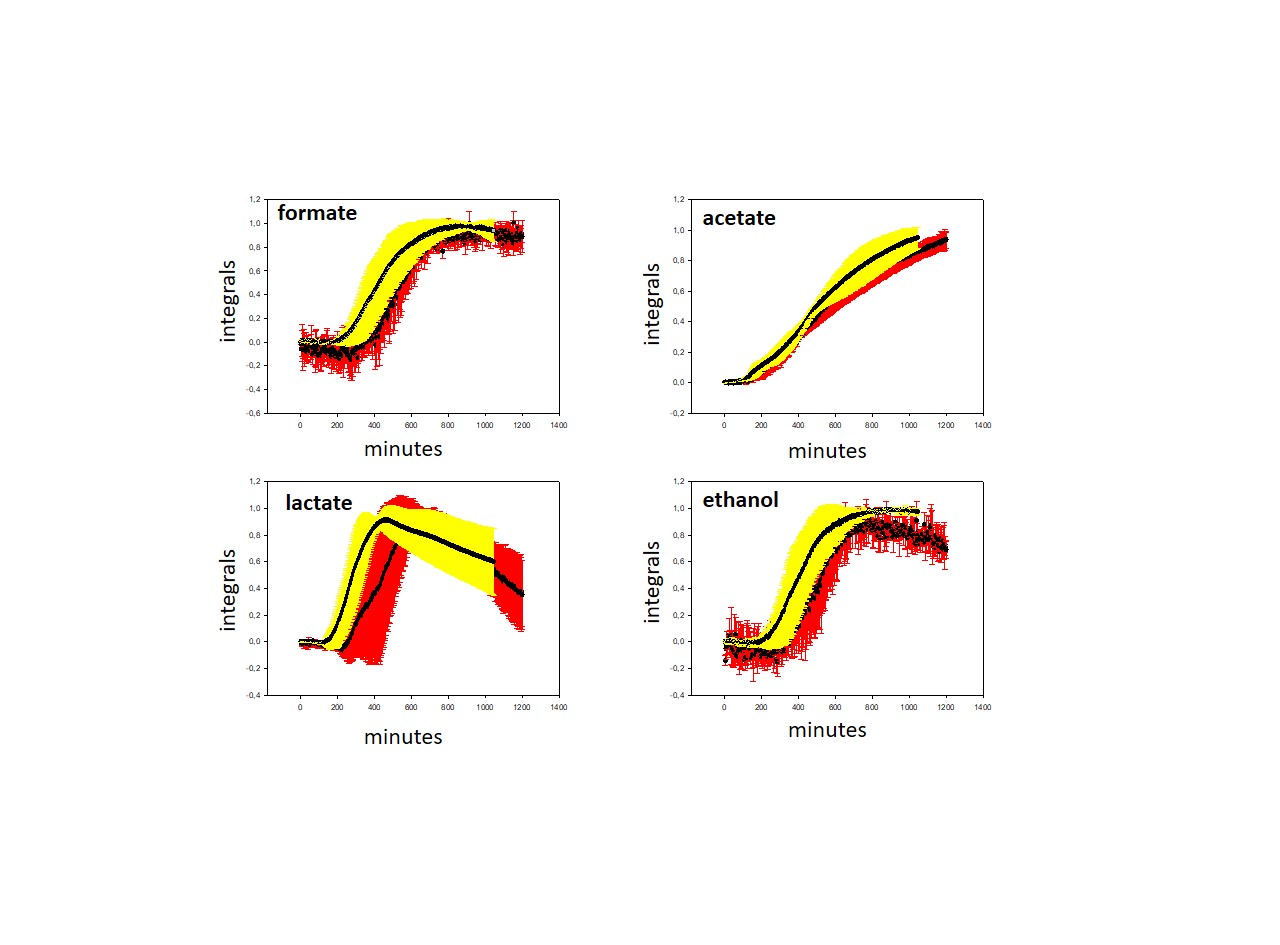
**

**Figure S4**. Secretion curves of formate, acetate, lactate and ethanol at different KPi/BHI v/v ratios observed for S. aureus. Errors are reported as standard deviations over three experiments and are shown in red for 5% and in yellow for 10% BHI


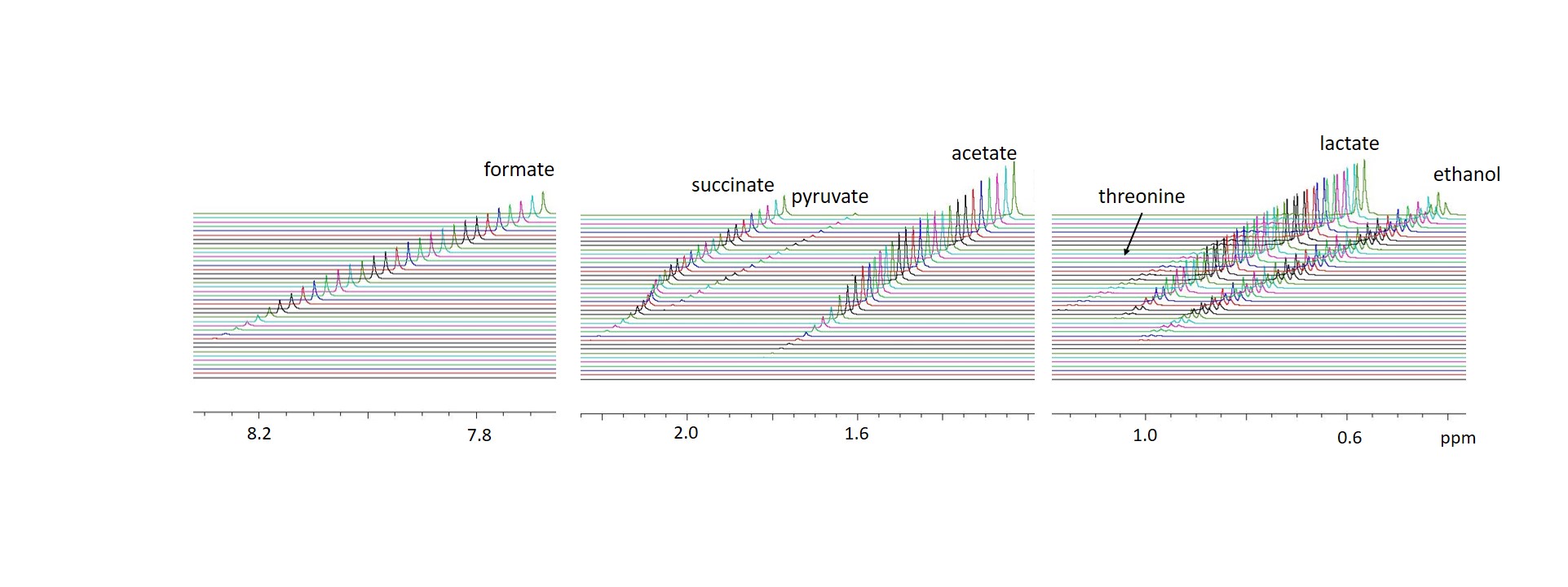


**Figure S5**. Series of 1D ^1^H NMR spectra recorded at 500MHz during 20 hours of *E. coli* glucose fermentation in the NMR tube. Spectra recorded at time intervals of 30 min are plotted with an horizontal offset of 0.02 ppm. The most abundant secreted metabolites are labelled.

Shift from aerobic to anaerobic metabolism within the NMR tube

A shift from aerobic to anaerobic metabolism within the NMR tube became evident when comparing the spectra of a cell culture after 30 minutes, 4 hours and 24 hours of incubation. This transition was marked by an increase in lactate NMR signals over time with a simultaneous decrease of the pyruvate NMR signal (Figure S6B, III–V). This shift to the anaerobic metabolism was expected considering the restricted surface area of the NMR sample.

To test whether the observed signals originated from secreted metabolites and not from highly concentrated intracellular molecules, aliquots of a bacterial cell culture were taken immediately after overnight growth. One of them was filtered with 0.22μm cutoff filter to remove all bacterial cells. Filtered and not filtered bacterial samples were immediately transferred to NMR tubes and ^1^H NMR spectra were recorded immediately and after six hours of incubation. If the NMR signals were due to intracellular metabolites, differences in the NMR spectra of filtered and not filtered samples were to be expected. However, no spectral differences were observed between the spectra recorded at time zero and six hours later (Figure S6A, I, and II), indicating that NMR metabolites signals derive from extracellular molecules only.


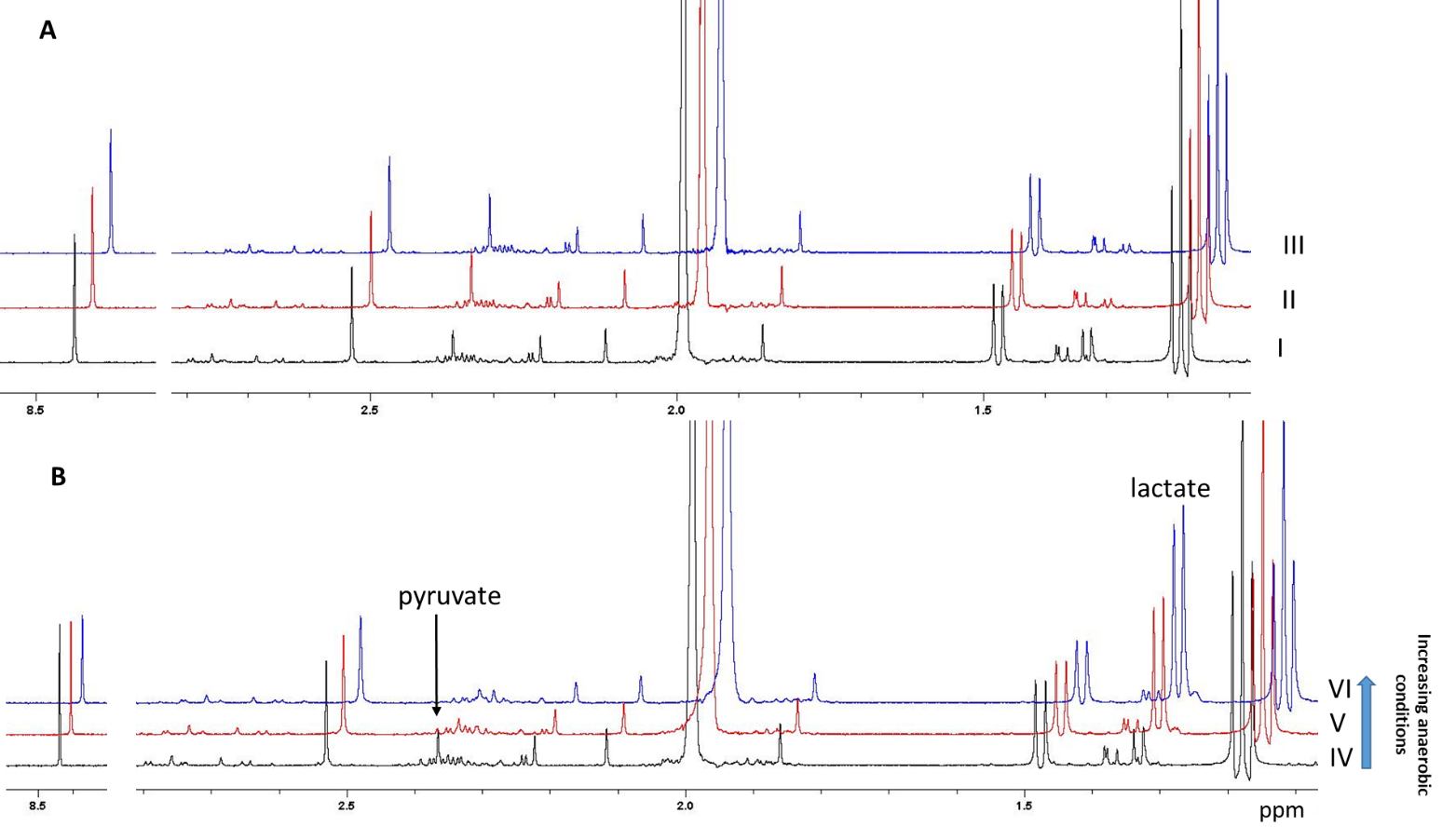


**Figure S6.** A) 1D ^1^H NMR spectra of an overnight E. coli cell culture grown in m.m acquired immediately before (I) and after filtering with a 0.22 μm filtration device (II). An additional spectrum was recorded after 6 hours in the NMR tube (III). B) 1D ^1^H NMR spectra of an overnight E. coli cell culture grown in m.m acquired after 30 min (IV), 4 hours (V) and 20 hours (VI) of cell growth in the NMR tube (blue arrow identify the increasing anaerobic conditions; lactate and pyruvate are indicated). Spectra are plotted with a horizontal offset of 0.03 ppm.

**Viability test of NMR samples at the end of acquisition time**

To further investigate if the observed signals are related to secreted metabolites and not to intracellular metabolites released after cell lysis or to leakage, viability tests were performed on samples extracted from the NMR tube at the end of NMR acquisition. Viability tests indicated that these samples contained viable E. coli cells (Figure S7).

**Figure S7**. Viability tests performed on the NMR samples at the end of the NMR experiments of E. coli (grey, 20 h), E. coli grown in the presence of kanamycin 10 μM (striped blue, 60 h) or ampicillin 0.24 μM (blue, 24 h) added to the m.m.

**NMR-based cell integrity test**

When E. coli switches from oxidative to fermentative pathway, the flow of metabolites through the Krebs cycle decreases and consequently 2-oxo-glutarate accumulates^2^. Signals of 2-oxo-glutarate are easily recognizable in the NMR spectrum acquired with the supernatant of a 10mL cell culture clarified lysate (Figure S8, black line). A rough estimate of its concentration was performed from the NMR signal at 3.05 ppm, indicating an intracellular concentration around 0.12 mM, in line with literature data ^2,3^.

Signals of 2-oxo-glutarate, the most abundant intracellular metabolite in cells growing under anaerobic conditions, were used as probe to follow cell lysis since 2-oxo-glutarate does not cross the cell membrane. Its signals were visible in the spectrum of a lysed 800uL culture grown in the NMR tube (blu spectrum in figure S8), while they were absent from the spectrum recorded at the end- point of the NMR acquisition of secretion curve (red spectrum in figure S8).

The absence of these signals in the intact culture (red spectrum) grown in the same conditions shows that most cells are intact and secreting metabolites. The minimum detectable concentration of oxo-glutarate is determined by spectral noise, which is 7 ± 2 μM in our experimental conditions.

In this respect, we estimated that less than 6% of intracellular 2-oxo-glutarate leaked out, further indicating that most of the cells remained intact.


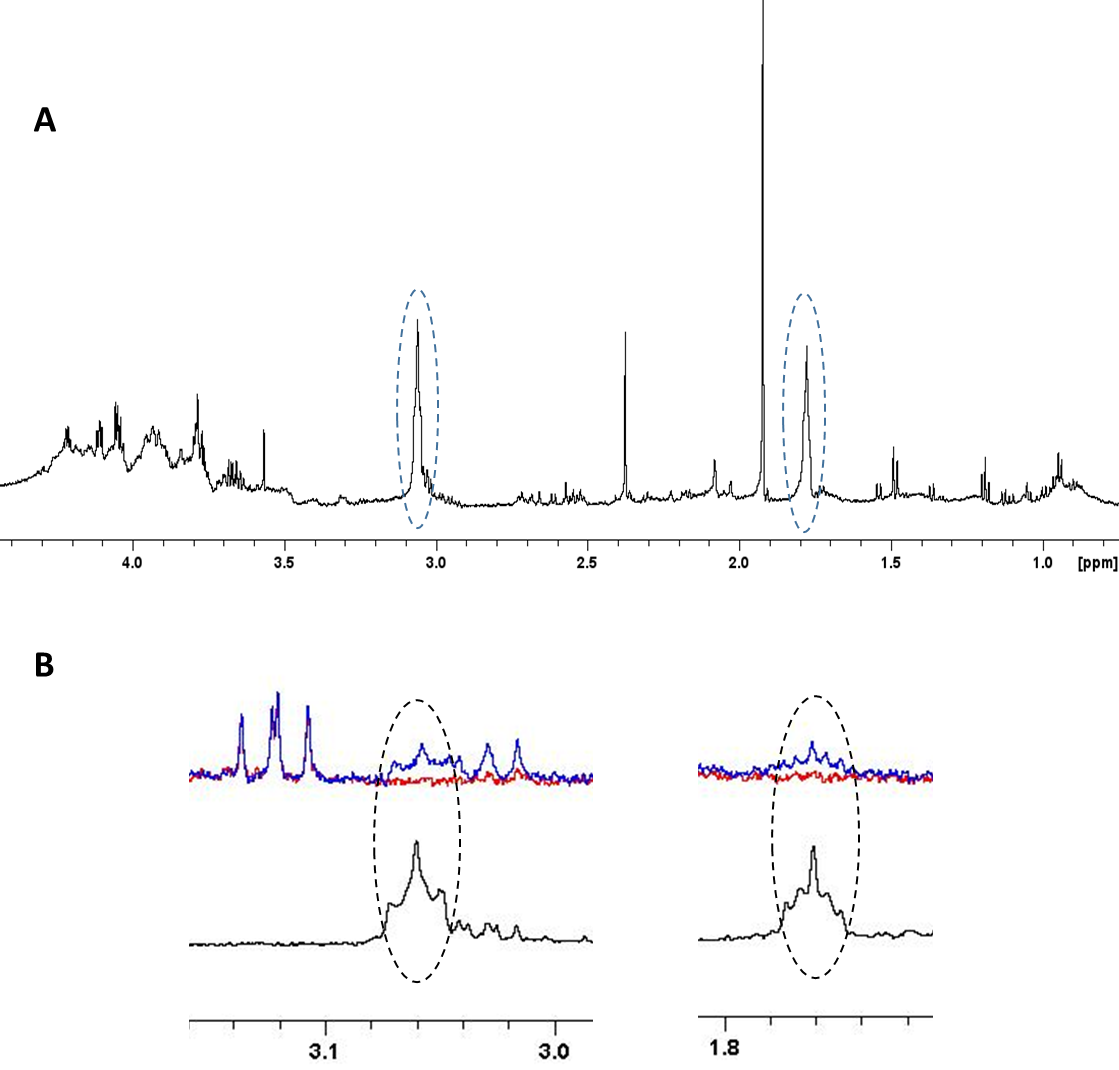


**Figure S8**. 1D 1H NMR spectra, acquired at 600 MHz, of intact (red) and a lysed (blue) 24-hour cell culture grown in the NMR tube (800 μl). The NMR spectrum of the supernatant of a 10 ml lysate from cells grown under the same anaerobic conditions as in the NMR tube is reported as a reference in panels A and B (black). The signals assigned to CH2 of 2-oxo-glutarate are circled. The experiments were repeated three times with independent cultures and acquisitions, yielding similar results. Here the result of one replica is reported for clarity reasons.


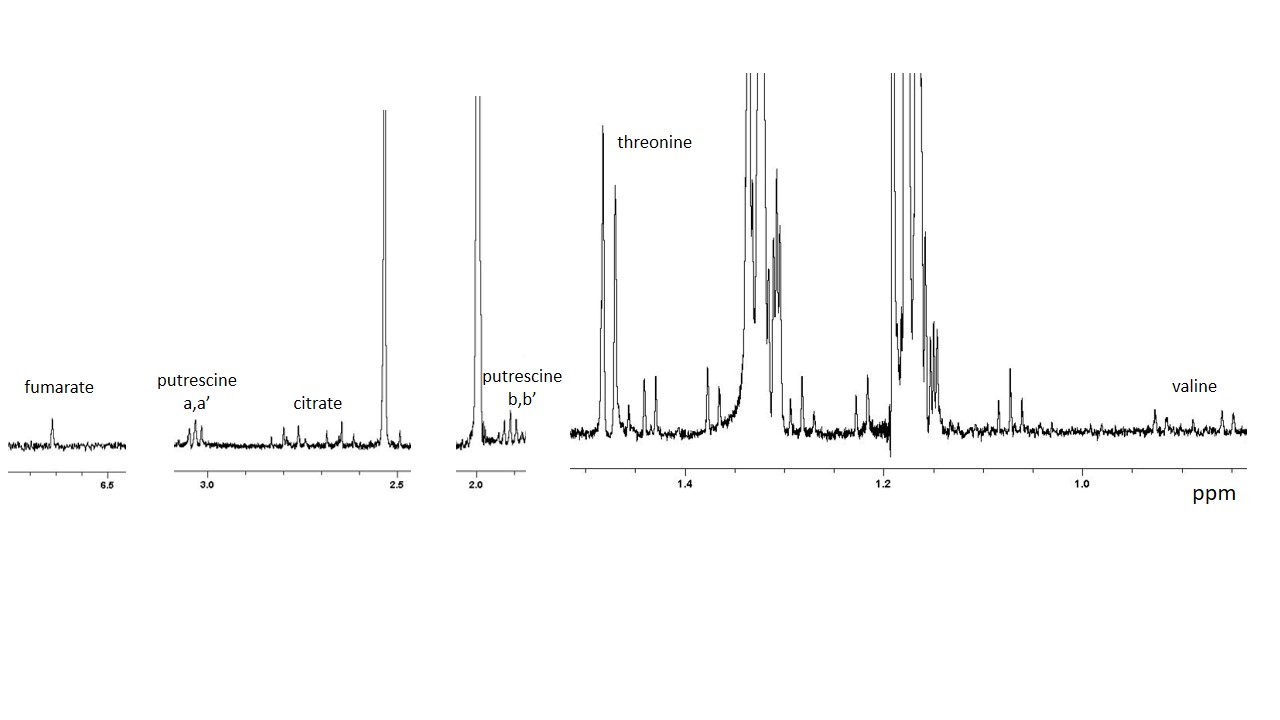


**Figure S9**. 1D ^1^H NMR spectrum recorded at 600MHz after 20 hours of *E. coli* glucose fermentation. Assignments are reported only for the less abundant secreted metabolites.


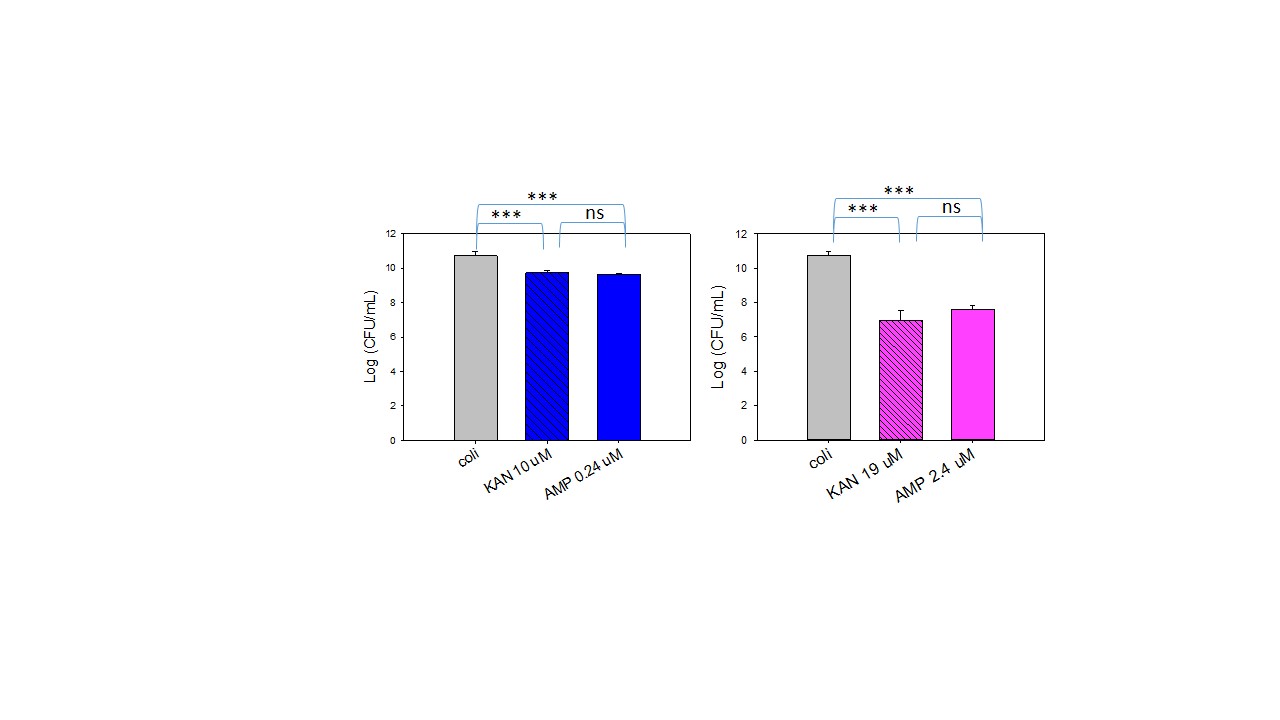


**Figure S10**. Viability tests (CFU counts) after 24 hours of exposure to 10 -19 μM kanamycin (stripped blue and pink bars) and 0.24-2.4 μM ampicillin (blue and pink plein bars) are shown in the inset. Differences between treated and untreated E. coli were evaluated using one-way analysis of variance (ANOVA) followed by Bonferroni post hoc testing. All statistical analyses were performed with a 95% confidence level; therefore, p-values ≥ 0.05 were considered not statistically significant. The Bonferroni t-test revealed statistically significant differences (***, p<0.001) for all pairwise comparisons, except for the direct comparison, both at lower and higher concentrations, between CFU counts which showed no significant difference (ns, p > 0.05).


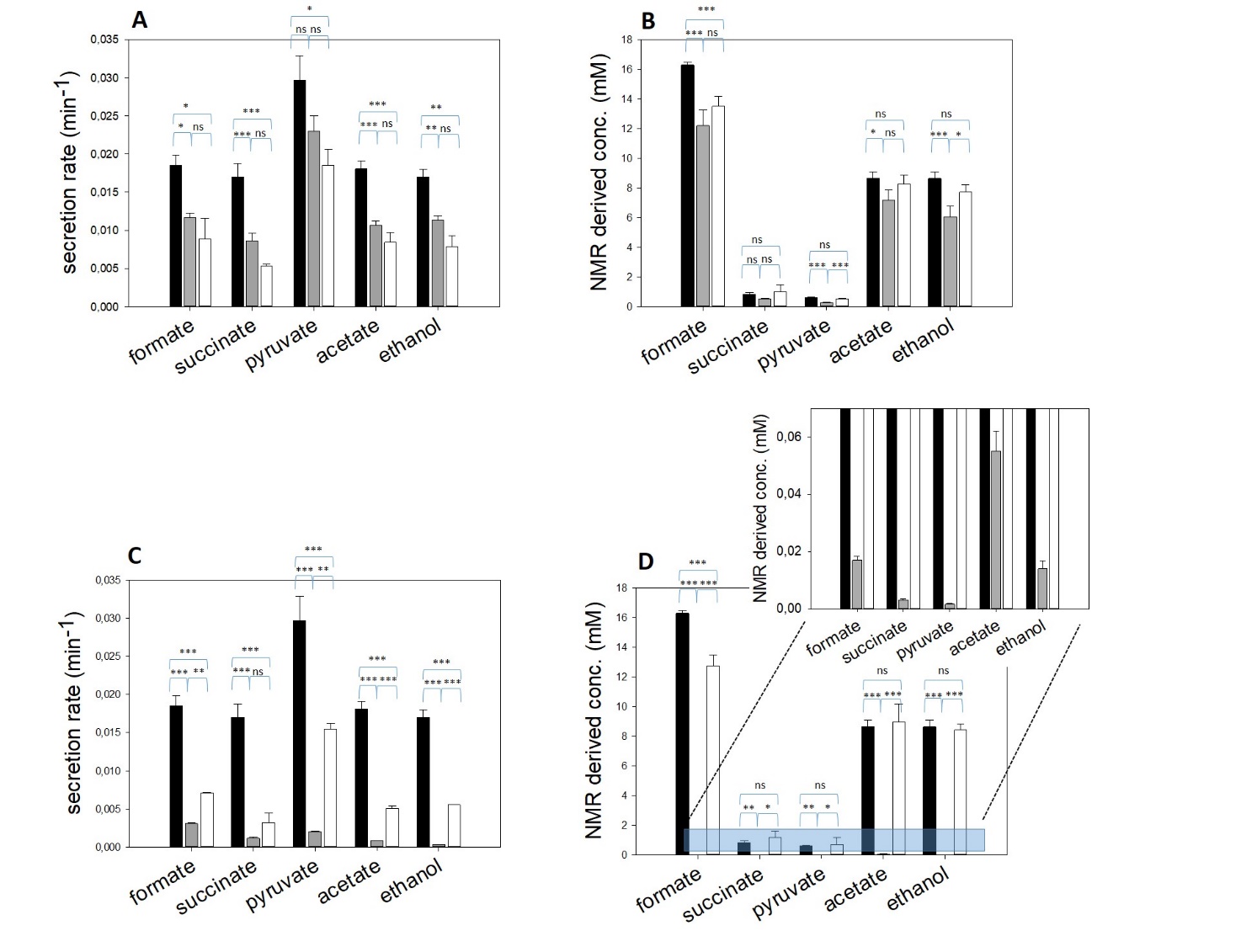


**Figure S11**. Secretion rates (A,C) and end point concentrations (B,D) for E. coli growing alone (black) or in the presence of kanamycin 10 μM (grey) and ampicillin 0.24 μM (white) (A,B), kanamycin 19 μM (grey) and ampicillin 2.4 μM (white) (C,D). Differences between treated samples and untreated E. coli were evaluated using one-way analysis of variance (ANOVA) followed by Bonferroni post hoc testing. All the comparisons passed ANOVA test. The Bonferroni t-test results are reported in figure as: ns p>0.05, * p< 0.05, ** p<0.01, *** p <0.001.

**Figure S12**. Growth curve of untreated E. coli (black) and in presence of kanamycin 10 μM (grey) and ampicillin 0.24 μM (dark grey). Data are presented in logarithmic scale as average of 3 independent measurements.


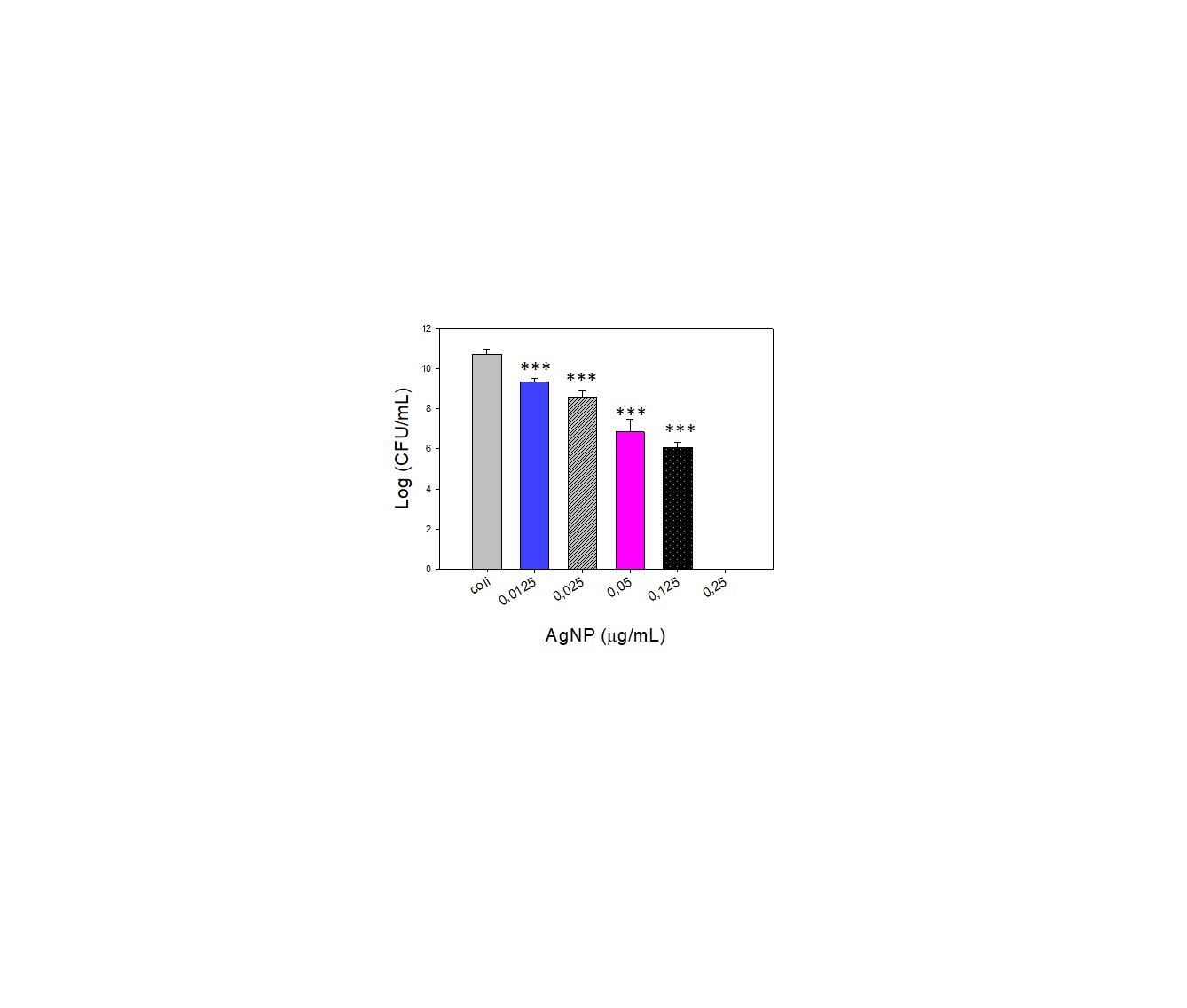


**Figure S13**. Colony forming unit in logarithmic scale of E. coli in the presence of different amounts of AgNP. CFU are reported for E. coli alone (plain grey) and in the presence of increasing amounts of AgNP. A CFU reduction by 3 Log_10_ (blue), 5 Log_10_ (pink), for an intermediate amount (stripped) and for higher amounts further decreasing CFU (black) was observed. MIC was evaluated at 0,25 μg/mL. Data are presented as mean ± standard deviation. Differences between treated samples and untreated E. coli were evaluated using two-tailed Student’s t test; ***, p<0.001


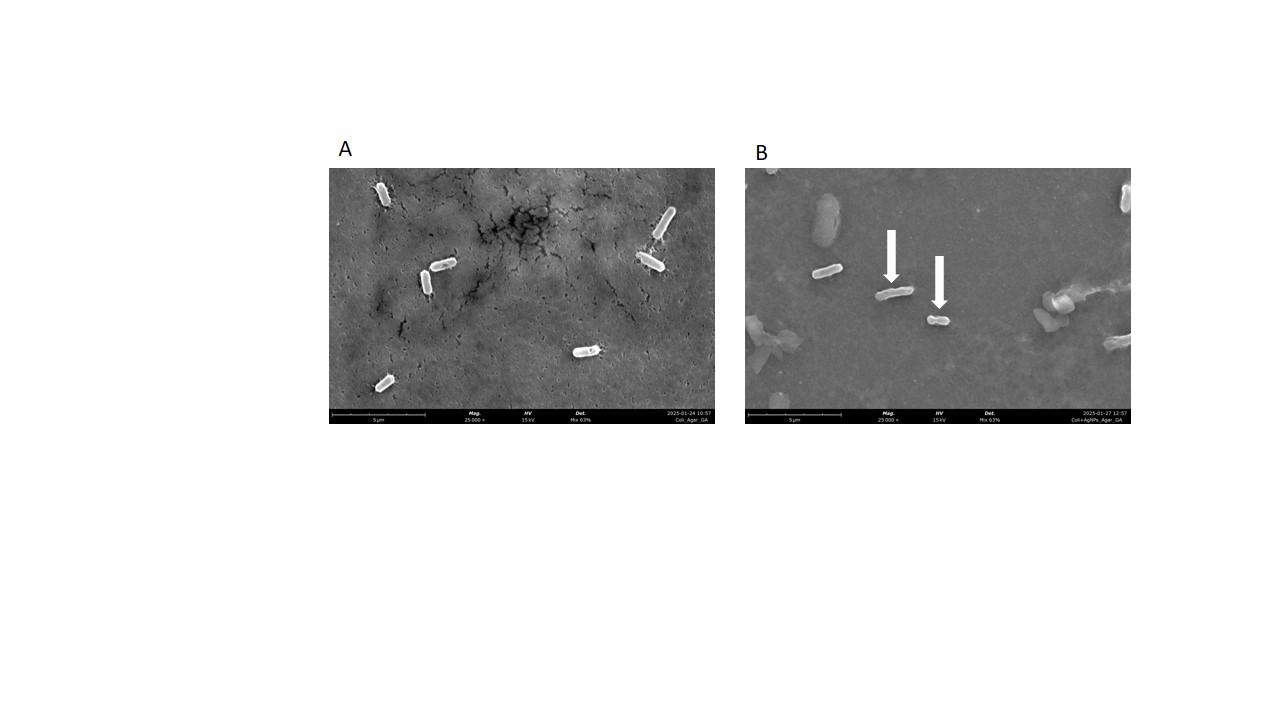


**Figure S14**. SEM images of E. coli alone (A) or incubated with 0,05 μg/ml AgNP (B) for 24 hours. Arrows indicated morphological variated cells. Magnification 25000 x

**Table S1**. Antibacterial agents concentration selected for viability tests in E. coli and S. aureus

| **Antibacterial agent** | **Concentration** |
| --- | --- |
| Kanamycin | 0.063, 0.63, 6.3, 10, 19, 63, 630 μM |
| Ampicillin | 0.024, 0.24, 2.4, 24, 240 μM |
| Caprylic acid | 0.4, 4 mM |
| IRGASAN | 0.0001, 0.0005, 0.001 mg/L (E. coli), 0.000005 and 0.00001 mg/L (S. aureus) |
| AgNP | 0.0125, 0.025, 0.05, 0.125, 0.25 μg/mL |
| GL NP | 10, 50, 100 μg/L GL NP |

|  | **E. Coli** | **10μM kan** | **19μM kan** | **0.24 μM amp** | **2.4 μM amp** | **0.0005 mg/L IRG** | **0.001 mg/L IRG** | **0.4mM CA** | **4mM CA** | **T4** | **0,0125 μg/ml AgNP** | **0,025 μg/ml AgNP** |
| --- | --- | --- | --- | --- | --- | --- | --- | --- | --- | --- | --- | --- |
| **formate** | 16.29±0.19 | 12.20±1.1 | 0.014±1*10^-3^ | 14.07±0.23 | 12.4±0.36 | 0.49±0.25 | - | 15.6±0.42 | 16.23±0.21 | 14.82±2.1 | 13.9±0.79 | 13.9±1.13 |
| **succinate** | 0.84±0.11 | 0.54±0.03 | 3.15*10^-3^±3.5*10^-4^ | 0.81±0.15 | 1.16±0.44 | 0.036±0.02 | - | 0.81±0.08 | 0.63±0.09 | 1.40±0.26 | 1.09±0.08 | 1.01±0.04 |
| **pyruvate** | 0.60±0.06 | 0.28±0.02 | 1.65*10^-3^±2.1*10^-4^ | 0.53±0.03 | 0.82±0.3 | 0.043±0.003 | - | 0.55±0.02 | 0.54±0.02 | 0.51±0.08 | 0.44±0.07 | 0.69±0.05 |
| **acetate** | 8.65±0.44 | 7.17±0.71 | 0.055±7*10^-3^ | 8.11±0.22 | 8.96±1.2 | 0,57±0.11 | 0.30±0.04 | 8.32±0.45 | 9.34±0.52 | 9.21±0.53 | 8.14±0.14 | 8.23±0.42 |
| **ethanol** | 8.63±0.44 | 6.06±0.70 | 0.014± 2.8*10^-3^ | 7.87±0.38 | 8.4±0.43 | - | - | 8.14±0.22 | 8.56±0.11 | 9.28±0.86 | 8.02±0.14 | 8.11±0.28 |

**Table S2**. E. coli exo-metabolites end-point concentration, expressed in mM, determined by NMR in presence of kanamycin 10-19 μM and ampicillin 0.24, 2.4 μM, 0.0005 and 0.001 mg/L IRG, 0.4 and 4 mM Caprilic acid, T4 silicon tube, 0.0125 and 0.025 AgNPs μg/mL

**Table S3**. Estimate of secreted metabolite moles per single cell for untreated and treated E. coli. Moles were estimated on the basis of NMR derived concentration rescaled on the total number of bacterial cells contributing over measuring time (10^11^ for E. coli in the absence and 10^9^ and 10^8^ for E. coli in the presence of ampicillin and kanamicin, respectively). Values are reported as mean ± standard deviation of 3 independent measurements.

| **Molecule** | **Untreated E. coli**  **(10^-15^ mol)** | **+ 10μM kanamycin**  **(10^-15^ mol)** | **+ 0.24 μM ampicillin**  **(10^-15^ mol)** |
| --- | --- | --- | --- |
| formate | 0,13±0,0015 | 97,6±8,8 | 11,25±0,18 |
| succinate | 0,0067±0,0009 | 4,32±0,24 | 0,64±0,12 |
| pyruvate | 0,0048±0,00048 | 2,24±0,16 | 0,42±0,024 |
| acetate | 0,069±0,0035 | 57,36±5,68 | 6,48±0,18 |
| ethanol | 0,069±0,0035 | 48,48±5,6 | 6,29±0,30 |

**Table S4**. S. aureus exo-metabolites end-point concentration (mM) determined by NMR in presence of 10, 50, 100 GL-NPs μg/L, 0.05, 0.125, 0.25 AgNPs μg/mL, 0.005*10^-3^ and 0.1*10^-3^ mg/L IRG

| **Metabolite** | **S. Aureus** | **10 μg/L GL-NP** | **50 μg/L GL-NP** | **100 μg/L GL-NP** | **0.05 μg/L AgNP** | **0.125 μg/L AgNP** | **0.25 μg/L AgNP** | **0.005*10^-3^ mg/L IRG** | **0.1*10^-3^ mg/L IRG** |
| --- | --- | --- | --- | --- | --- | --- | --- | --- | --- |
| **Formate** | 0.25±0.02 | 0.22±0.03 | 0.20±0.08 | 0.25±0.01 | 0.23±0.01 | 0.35±0.1 | 0.23±0.01 | - | - |
| **Acetate^1^** | 0.53±0.03 | 0.60±0.08 | 0.52±0.007 | 0.52±0.007 | 0.47±0.02 | 0.37±0.18 | 0.48±0.02 | 0.28±0.007 | 0.13±0.007 |

^1^ The acetate concentration should be considered a lower-limit estimate due to partial overlap with nutrient resonances, which are possibly consumed over time.

(1) Ferreira, M. T.; Manso, A. S.; Gaspar, P.; Pinho, M. G.; Neves, A. R. Effect of Oxygen on Glucose Metabolism: Utilization of Lactate in Staphylococcus Aureus as Revealed by In Vivo NMR Studies. *PLOS ONE* **2013**, *8* (3), e58277. https://doi.org/10.1371/journal.pone.0058277.

(2) *Overcoming Fluctuation and Leakage Problems in the Quantification of Intracellular 2-Oxoglutarate Levels in Escherichia coli*. https://doi.org/10.1128/AEM.05257-11.

(3) Thomas, A. D.; Doelle, H. W.; Westwood, A. W.; Gordon, G. L. Effect of Oxygen on Several Enzymes Involved in the Aerobic and Anaerobic Utilization of Glucose in Escherichia Coli. *J. Bacteriol.* **1972**, *112* (3), 1099–1105. https://doi.org/10.1128/jb.112.3.1099-1105.1972.
